# *In*-*situ* RhoA editing *via* heparinylated LNP-microsphere system for rheumatoid arthritis treatment

**DOI:** 10.1186/s12951-026-04040-x

**Published:** 2026-01-23

**Authors:** Yingchun Zhu, Lei Wang, Yingying Wei, Guanrong Li, Zheyuan Shi, Dianqing Wang, Qiang Wang, Liheng Wang, Weibing Si, Xing Yang

**Affiliations:** 1 Department of Orthopaedic Surgery, The First Affiliated Hospital of Ningbo University, Ningbo, 315010 P. R. China; 2 Department of Orthopedics, The First Affiliated Hospital of Wannan Medical College, Yijishan Hospital of Wannan Medical College, Wuhu, 241001 P. R. China; 3 Department of Orthopedics, The Affiliated Suzhou Hospital of Nanjing Medical University, Suzhou Municipal Hospital, Suzhou, 215000 P. R. China; 4 Gusu School of Nanjing Medical University, Suzhou, 215000 P. R. China; 5 Department of Orthopedics, Shanghai Key Laboratory for Prevention and Treatment of Bone and Joint Diseases, Shanghai Institute of Traumatology and Orthopaedics, Ruijin Hospital, Shanghai Jiao Tong University School of Medicine, Shanghai, 200025 P. R. China

**Keywords:** Rheumatoid arthritis, mRNA strategy, Macrophage, Microsphere, Local treatment

## Abstract

Macrophages play a pivotal role in modulating inflammation and osteoclastogenesis during the progression of rheumatoid arthritis (RA). Although local mRNA-mediated gene editing offers high precision and efficiency, there remains a lack of robust strategies tailored for effective editing of synovial macrophages. Here, we identified RhoA as a macrophage target *via* bioinformatics, validated its therapeutic potential, and built a heparinylated LNP-microsphere system (hLNP-RhoA^−/−^@MS) to replace PEGylated LNPs for in situ RhoA editing. Specifically, a low-molecular weight heparin-cholesterol conjugate was employed in place of PEG during LNP formulation. Substituting PEG with heparin on the LNP surface markedly enhanced the transfection efficiency of delivered mRNA/sgRNA. In parallel, integration of the microsphere system substantially improved LNP stability and intra-articular retention. In vitro RNA-seq analysis revealed broad and significant suppression of multiple key inflammation-related signaling pathways following RhoA editing. In the RA mouse model in vivo, hLNP-RhoA^−/−^@MS conferred pronounced therapeutic benefits, including attenuation of joint inflammation, inhibition of cartilage and bone destruction, and remodeling of the immune microenvironment, thereby demonstrating strong anti-RA efficacy. Overall, we present a feasible, effective local mRNA editing strategy for precise modulation of synovial macrophages in RA.

**Graphical Abstract:**

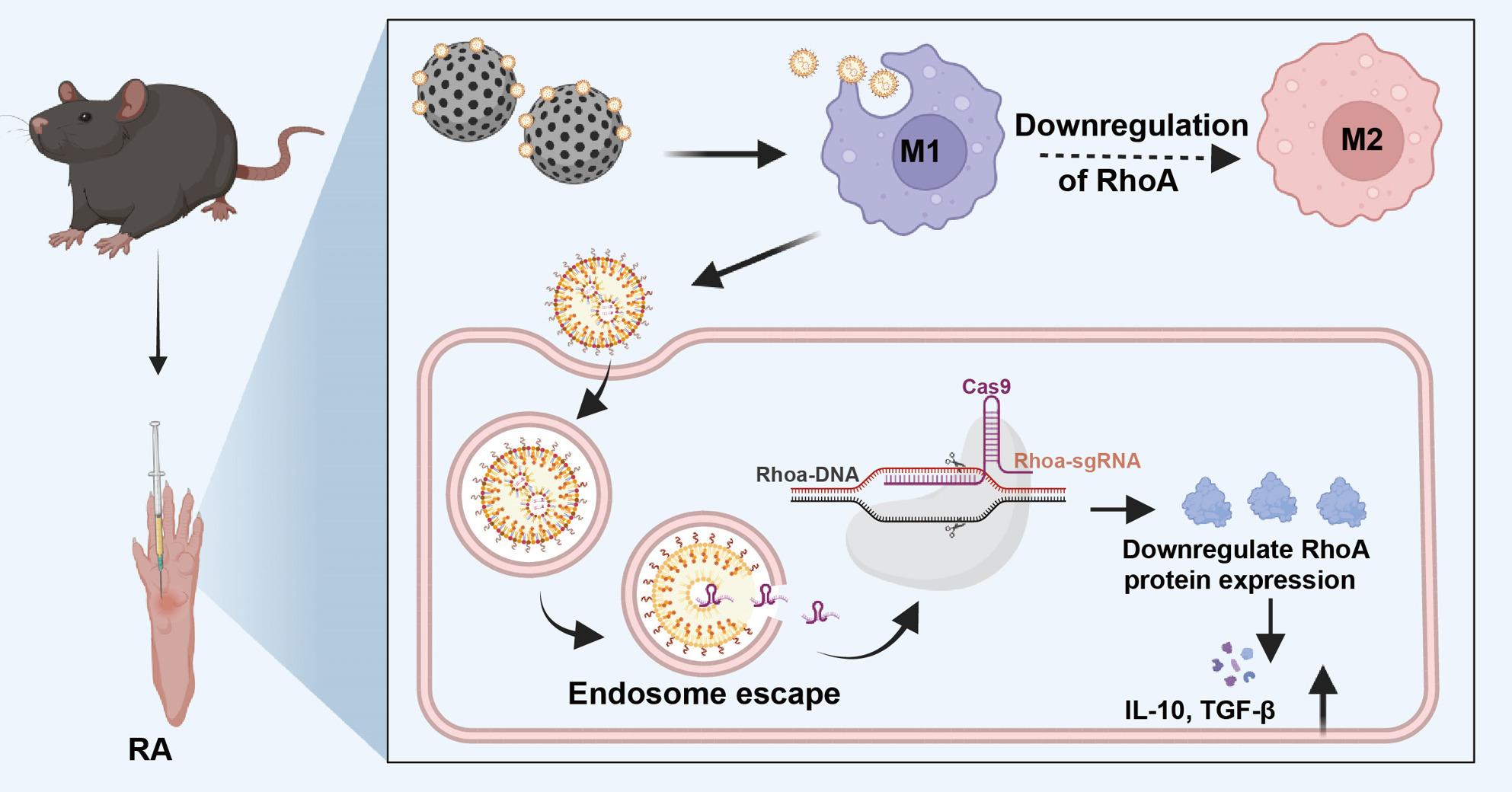

**Supplementary Information:**

The online version contains supplementary material available at 10.1186/s12951-026-04040-x.

## Introduction

Rheumatoid arthritis (RA) is a common autoimmune disease characterized by chronic synovial inflammation [1]. With disease progression, inflammation-driven synovial hyperplasia and erosive remodeling lead to continuous destruction of cartilage and bone, resulting in marked deterioration of quality of life [2]. Current therapies, including nonsteroidal anti-inflammatory drugs, immunosuppressants, and biologics, primarily act on downstream inflammatory pathways; although they alleviate symptoms and partially delay progression, they rarely prevent structural damage or arrest the disease at its source. Increasing evidence indicates that small GTPases, such as RhoA, occupy upstream regulatory positions in controlling the inflammatory phenotype of synovial macrophages and the invasiveness of synovial fibroblasts, rendering them promising targets for gene therapy [3]. Intra-articular, in situ gene editing directed at these upstream nodes may enable genuine disease modification.

Despite the impact of PEGylated lipid nanoparticles (LNPs) on systemic delivery of CRISPR mRNA platform, their performance in intra-articular delivery and in situ editing remains constrained [4]. The PEGylating imposes three principal limitations in the local editing: it weakens interactions with the synovial barrier and extracellular matrix, reducing tissue penetration; it diminishes uptake by key effector cells, such as synovial macrophages, limiting access of the editing machinery to diseased cells; and it increases the risk of sedimentation, activity loss, and cargo leakage during formulation and storage [5–7]. Increasing cationic components can enhance uptake and mitigate aggregation, but at the cost of elevated cytotoxicity and greater mRNA surface adsorption, thereby compromising cargo stability [8]. Accordingly, a PEG alternative is required that balances stability, penetration, and cellular uptake, and that is compatible with the inflammatory microenvironment of RA.

Low-molecular-weight heparin (LMWH) is a negatively charged sulfated polysaccharide that has been investigated in RA and can exert anti-inflammatory effects through binding to multiple inflammatory mediators [9]. Its high affinity for inflammatory mediators and related receptors may establish a favorable biointerface within the joint microenvironment, a property not afforded by inert PEG. Hyaluronic acid (HA) hydrogel microspheres are widely used for joint lubrication and inflammation mitigation, and have been validated as carriers that enhance the stability, prolong the intra-articular retention, and enable sustained release of nanotherapeutics. On this basis, combining heparinized surface modification with HA microsphere encapsulation is expected to preserve the colloidal stability required by LNPs while improving synovial tissue penetration and macrophage uptake, thereby enabling precise local delivery.

In this study, public clinical datasets were analyzed to identify RhoA as a representative small GTPase target in RA. A heparinized LNP-microsphere composite (hLNP@MS) was then constructed to deliver CRISPR/Cas9 mRNA and sgRNA targeting RhoA for intra-articular, in situ gene editing (Scheme 1). To our knowledge, this is the first demonstration of replacing PEGylation with heparinization in mRNA-LNP design, addressing the stability-penetration-cellular uptake trade-offs in the RA joint environment. Encapsulation of hLNPs within HA hydrogel microspheres further increased local retention and on-site efficacy. In vitro, hLNPs enhanced RhoA editing in macrophages and reversed inflammatory phenotypes; in vitro and in vivo evaluations demonstrated that CRISPR/Cas9-mediated RhoA editing significantly ameliorated RA pathology. Overall, the hLNP@MS platform provides a stable, targeted, and efficient strategy for in situ gene editing in RA, with notable translational potential.

**Fig. 1:**
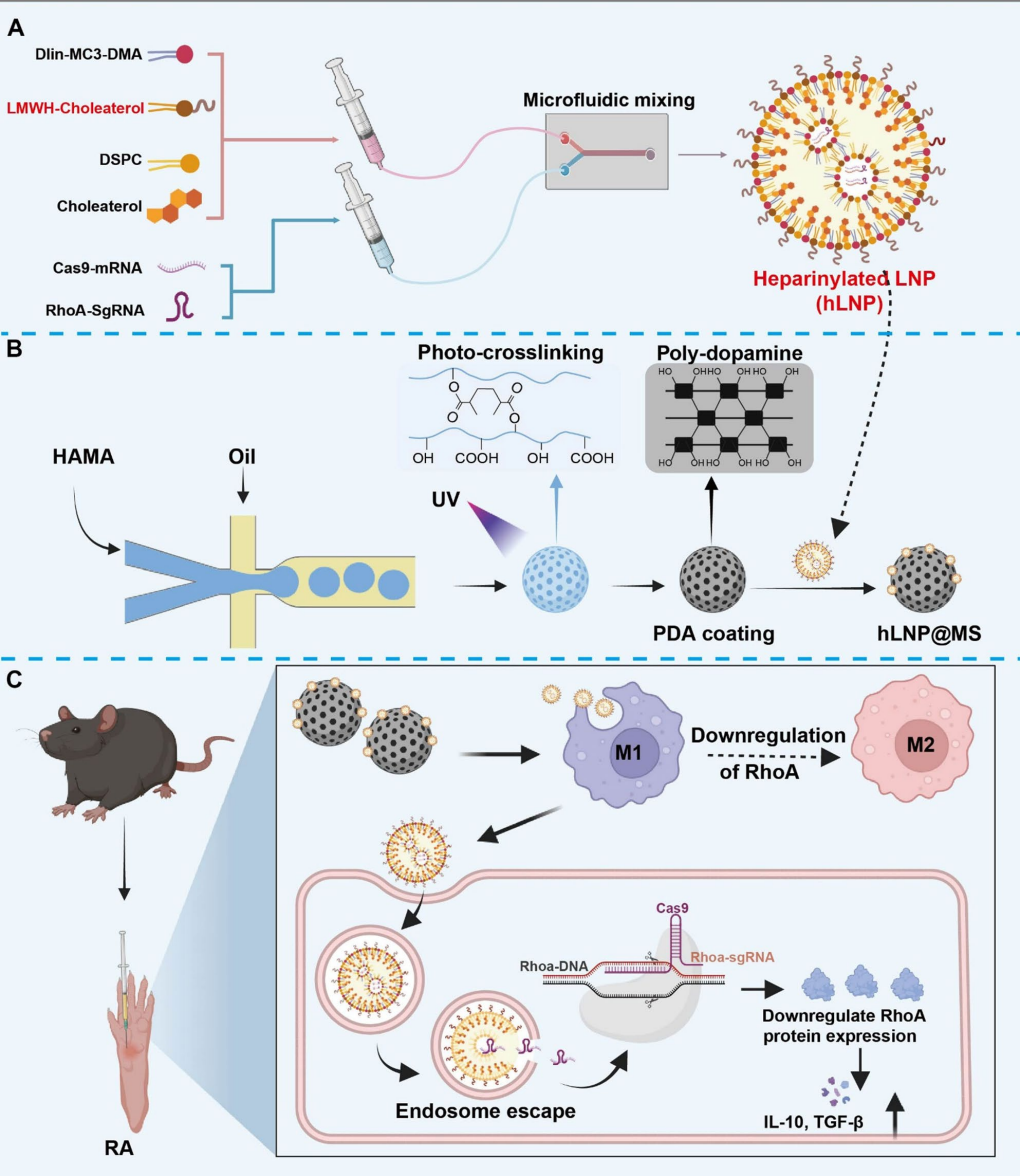
Preparation of hLNP-RhoA^−/−^@MS and therapeutic mechanism in RA: (**A**) Incorporation of LMWH–cholesterol into a standard LNP formulation and process to construct hLNPs. (**B**) Fabrication of HAMA hydrogel microspheres via microfluidics, followed by polydopamine (PDA) coating; hLNPs are then complexed with the PDA-coated microspheres through electrostatic and other physical interactions. (**C**) After intra-articular injection, hLNP-RhoA^−/−^@MS mediates macrophage targeting and RhoA gene editing in situ

## Results and discussion

### Preparation of heparinylated LNP (hLNP)

Traditional liposomes, including LNPs, have been primarily engineered for prolonged systemic circulation [10]. Incorporation of PEG reduces opsonization and immune recognition, thereby extending blood residence time and improving colloidal stability and dispersibility during LNP preparation [11]. However, conventional PEGylated LNPs are suboptimal for delivery to immune cells, particularly when targeted engagement of tissue-resident macrophages is required. Here, we introduce a heparin-based alternative to PEG to generate heparinylated LNPs (hLNPs), enabling localized delivery of mRNA and gene therapies to macrophages.

**Fig. 2:**
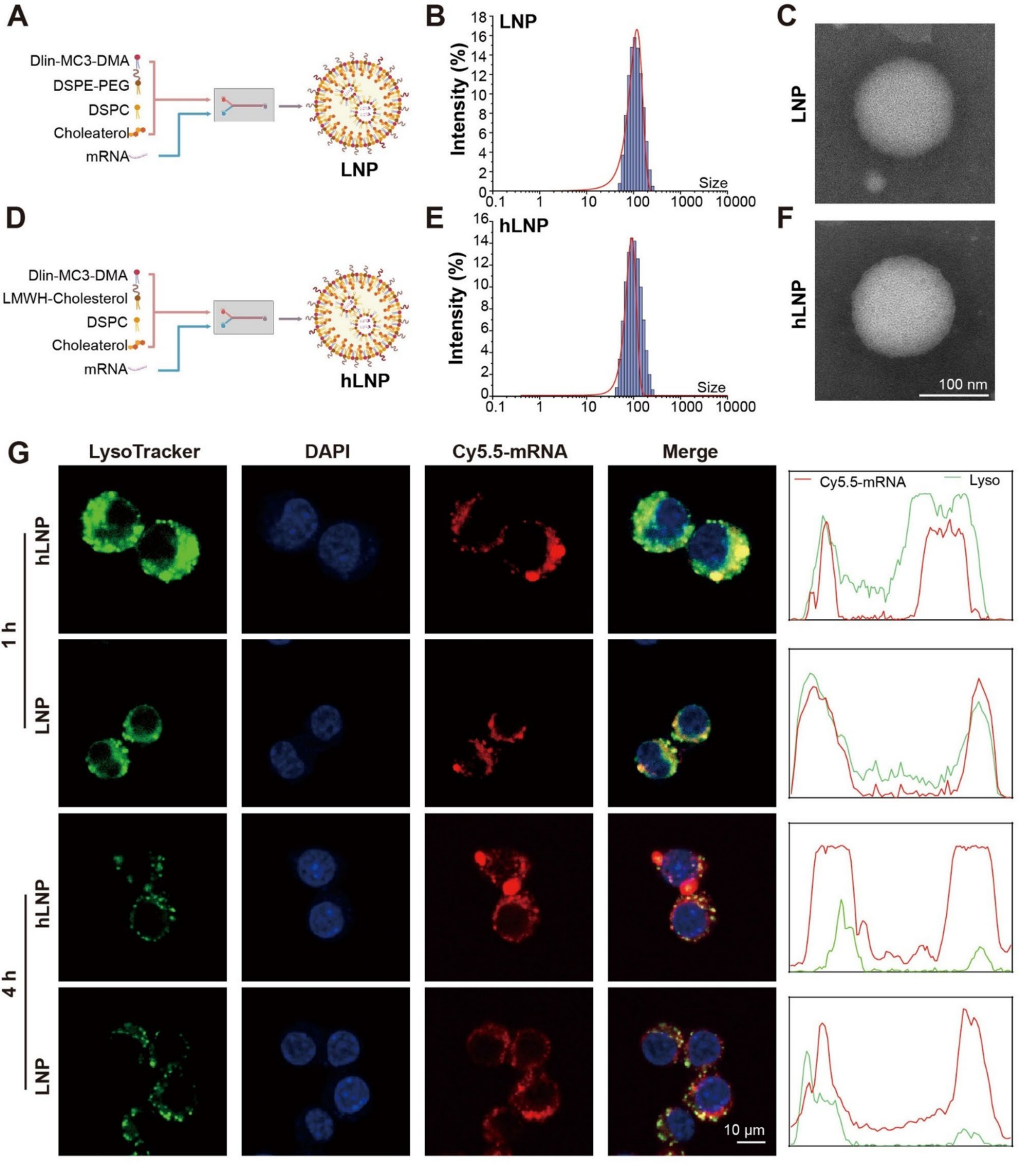
Preparation of hLNPs and assessment of cellular uptake: (**A–C**) Schematic of conventional LNP fabrication (**A**), size distribution (**B**), and TEM image (**C**). (**D–F**) Schematic of hLNP fabrication (**D**), size distribution (**E**), and TEM image (**F**). (**G**) Lysosomal colocalization analysis of Raw264.7 cells incubated with LNPs or hLNPs loaded with Cy5.5-mRNA; lysosomes were stained with LysoTracker Green

We retained the classical four-component formulation of LNPs, including ionizable lipid, helper phospholipid, cholesterol, and hydrophilic lipid components [12]. The same microfluidic device and preparation parameters were used for the fabrication of hLNPs. We chose low molecular weight heparin (LMWH) as a substitute for PEG not only because it has a longer half-life and higher safety, but also because its molecular weight is closer to that of PEG, resulting in less impact on the physical properties of LNPs [9]. Low-molecular-weight heparin (LMWH) was selected as a PEG substitute based on its favorable half-life and safety profile, as well as its comparable molecular weight to PEG, which minimizes perturbations to LNP physicochemical properties [13]. To promote incorporation into the lipid phase, we employed a well-characterized cholesterol-conjugated LMWH (LMWH-cholesterol), thereby increasing hydrophobicity and assembly competence (Fig. 2A and D). The molar fraction of LMWH-cholesterol was matched to that of the PEG-lipid in conventional PEGylated LNPs (1.5% mol). Following identical microfluidic mixing, dilution, and ultrafiltration, we conducted an initial physicochemical characterization of LNPs and hLNPs. Dynamic light scattering (DLS) and transmission electron microscopy (TEM) were used to observe the morphological changes of LNPs [14]. As shown in Fig. 2B and E, LNPs and hLNPs exhibited almost identical particle size distributions, with an average diameter of approximately 100 nm. TEM images (Fig. 2C and F), also confirmed the DLS results regarding the similar particle sizes of LNPs and hLNPs. Zeta-potentials of LNPs and hLNPs were detected as well. Compared with PEGylated LNPs (~ −10 mV), hLNPs exhibit approximately twice the negative surface charge (~ − 20 mV). The higher negative charge enhances the colloidal dispersion stability of the hLNP system (Figure S1). Notably, both formulations presented uniform, spherical morphology without evident heterogeneity. The slightly larger diameters recorded by DLS reflect hydrodynamic sizing, as widely discussed in prior reports [15].

Nanoparticles such as LNPs are mainly taken up through lysosome-mediated endocytosis [16]. The most prominent feature of LNPs is the protonation property in weakly acidic environments, introduced by ionizable lipids, which induces the proton sponge effect and thereby accelerates lysosomal escape [17]. To ensure that hLNPs possess lysosomal escape capabilities similar to conventional LNPs, we further investigated the colocalization of hLNPs with lysosomes after cellular uptake using Raw264.7 monocytes. To accurately localize hLNPs, we introduced Cy5.5-mRNA (Scrambled control) as a model mRNA for fluorescent labeling. LysoTracker Green dye was used to stain the lysosomes. After co-incubation with LNPs for 1 and 4 h, Raw264.7 cells were stained and then observed under a laser confocal microscope. As shown in Fig. 2G, LNPs and hLNPs exhibited very similar lysosomal colocalization characteristics. After 1 h, their fluorescence largely overlapped with LysoTracker. In contrast, hLNPs exhibited stronger Cy5.5 fluorescence and more intense lysosomal staining. However, after 4 h, this overlap was markedly reduced. The degree of colocalization in the hLNPs group was lower than that in the LNPs group. It is demonstrated that the PEG component with LMWH not only yields higher transfection capability but also enhances lysosomal escape.

Moreover, to verify whether hLNPs might mediate stronger macrophage endocytosis through interactions with inflammatory factors, we selected a common inflammatory cytokine, TNF-α (1,000 pg/mL) in complete medium, and pre-incubated it with hLNPs for 2 h prior to assessing macrophage uptake; conventional LNPs were used as controls [18]. As shown in Figure S2, hLNPs exhibited higher uptake by Raw264.7 cells than LNPs. This indicates that, compared with conventional PEGylated LNPs, heparin-functionalized hLNPs display markedly enhanced macrophage uptake after preincubation in TNF‑α-containing medium. The effect is likely driven by a TNF‑α-dependent protein corona that promotes cellular binding and endocytosis.

PEGylation has long been employed to prolong nanoparticle circulation; indeed, PEGylated liposomes are commonly described as long-circulating formulations [19]. This pharmacokinetic benefit arises primarily because PEG reduces opsonization and recognition by macrophages and other immune cells [20]. While PEGylation facilitates tissue targeting under systemic administration, it is suboptimal for local delivery when macrophage engagement is desired. As a hydrophilic stealth polymer, PEG diminishes macrophage endocytosis [21]. Consequently, conventional PEGylated LNPs are poorly suited for macrophage-targeted interventions *via* intra-articular administration in RA.

PEG also contributes to LNP stability, and its complete removal can promote aggregation; thus, an alternative hydrophilic polymer is required. In this work, we selected LMWH as a PEG substitute because it confers PEG-like hydrophilicity and electrostatic stabilization, exhibits immunomodulatory activity with affinity for inflammatory mediators, and, critically, is more readily recognized by macrophages, thereby enhancing cellular uptake. Accordingly, heparinylation offers a rational strategy to improve local delivery of mRNA-loaded LNPs to macrophages.

### Bioinformatics analysis of clinic RNA-seq dataset of RA patients

After clarifying the selection of mRNA delivery tools for local therapy, the target genes for RA treatment were further investigated. Although target genes such as inflammatory cytokines have been preliminarily validated to play a certain role in RA therapy, the effect of inhibiting or activating a single factor on RA remains limited [17]. Moreover, in the complex pathogenesis of RA, the activation of inflammation is only one aspect; therefore, new targets should have the potential for multi-mechanistic regulation at a deeper level.

**Fig. 3:**
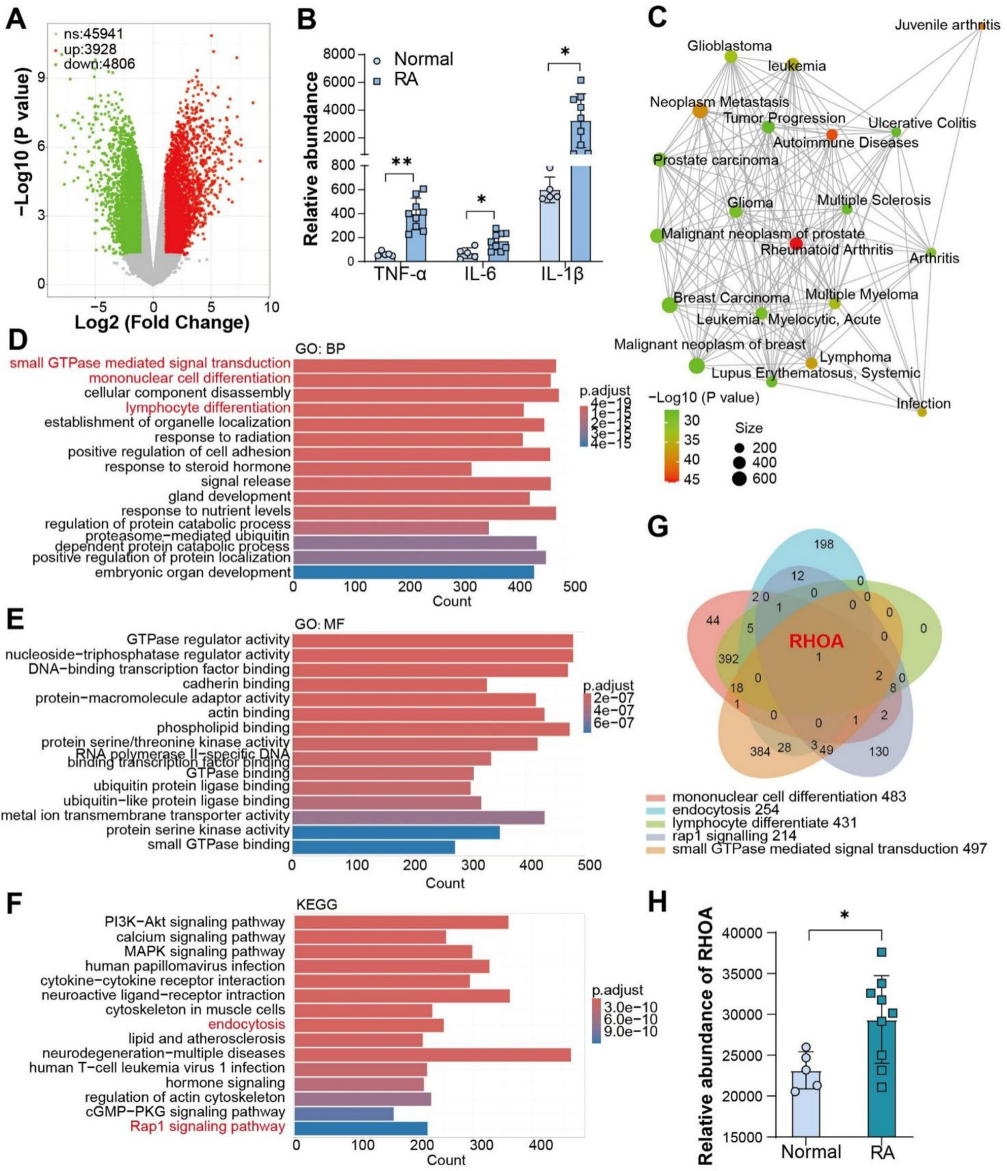
Transcriptomic profiling and functional enrichment analyses in RA patients versus healthy controls: (**A**) Volcano plot showing differentially expressed genes between RA patients and healthy controls; (**B**) Relative expression levels of inflammatory cytokines (TNF-α, IL-6, IL-1β) in RA versus controls; (**C**) Disease association analysis indicating that the differentially expressed genes are significantly enriched in multiple autoimmune diseases, particularly RA; (**D**) GO Biological Process (BP) enrichment analysis; (**E**) GO Molecular Function (MF) enrichment analysis; (**F**) KEGG pathway analysis; (**G**) Venn diagram showing that RHOA intersects multiple key immune-regulatory pathways; (**H**) Quantification demonstrating that RHOA expression is significantly higher in RA patients than in controls. (Data were analyzed by one-way ANOVA and post-hoc multiple comparisons, ns indicates no statistically significant difference, *P < 0.05, **P < 0.01, ***P < 0.001)

We performed differential expression analysis on RNA-seq data from RA patients and healthy individuals available in public database (GSE97779) [22]. The volcano plot revealed 3,928 significantly upregulated genes and 4,806 significantly downregulated genes (Fig. 3A). We then profiled the expression levels of inflammation-related factors and found that TNF-α, IL-6, and IL-1β were all significantly elevated in RA patients compared with controls (Fig. 3B), further confirming a markedly heightened inflammatory response within RA tissues. Disease association network analysis indicated that the differentially expressed genes are closely linked to multiple autoimmune diseases and cancers, with a particularly strong association with rheumatoid arthritis (Fig. 3C).

To clarify the functional characteristics of these genes, we conducted GO enrichment analysis on genes significantly upregulated in the RA transcriptome. Biological process (BP) analysis showed enrichment in immune-regulatory pathways, including small GTPase-mediated signal transduction, monocyte differentiation, and lymphocyte differentiation (Fig. 3D). Molecular function (MF) analysis indicated that these genes are primarily involved in GTPase regulation, DNA binding, ubiquitin ligase binding, and protein kinase activity (Fig. 3E), suggesting that RA pathogenesis is tightly connected to immune cell differentiation, signal transduction, and protein degradation. KEGG pathway analysis further showed enrichment in the PI3K-Akt, MAPK, cytokine-receptor interaction, and Rap1 signaling pathways (Fig. 3F).

Subsequent Venn analysis revealed that RhoA intersects across key pathways (including monocyte differentiation, lymphocyte differentiation, and small GTPase signaling), highlighting it as a central regulatory factor (Fig. 3G). Gene expression analysis confirmed a significant difference in RhoA abundance between the RA and control groups (Fig. 3H). Together, these findings suggest that RhoA, a pivotal member of the small GTPase family, may serve as a hub in the immune regulation and inflammatory responses of RA, representing a potential therapeutic target.

### *In vitro* RhoA editing analysis and preparation of hLNP-RhoA^−/−^

Bioinformatic analyses identified RhoA as a compelling target for gene therapy in RA. To interrogate its pathogenic role, we first performed preliminary in vitro studies using a pharmacological RhoA inhibitor. We then engineered a CRISPR/Cas9 system comprising Cas9 mRNA and a target-specific sgRNA, and delivered it *via* hLNPs for subsequent in vitro and in vivo evaluation.

**Fig. 4:**
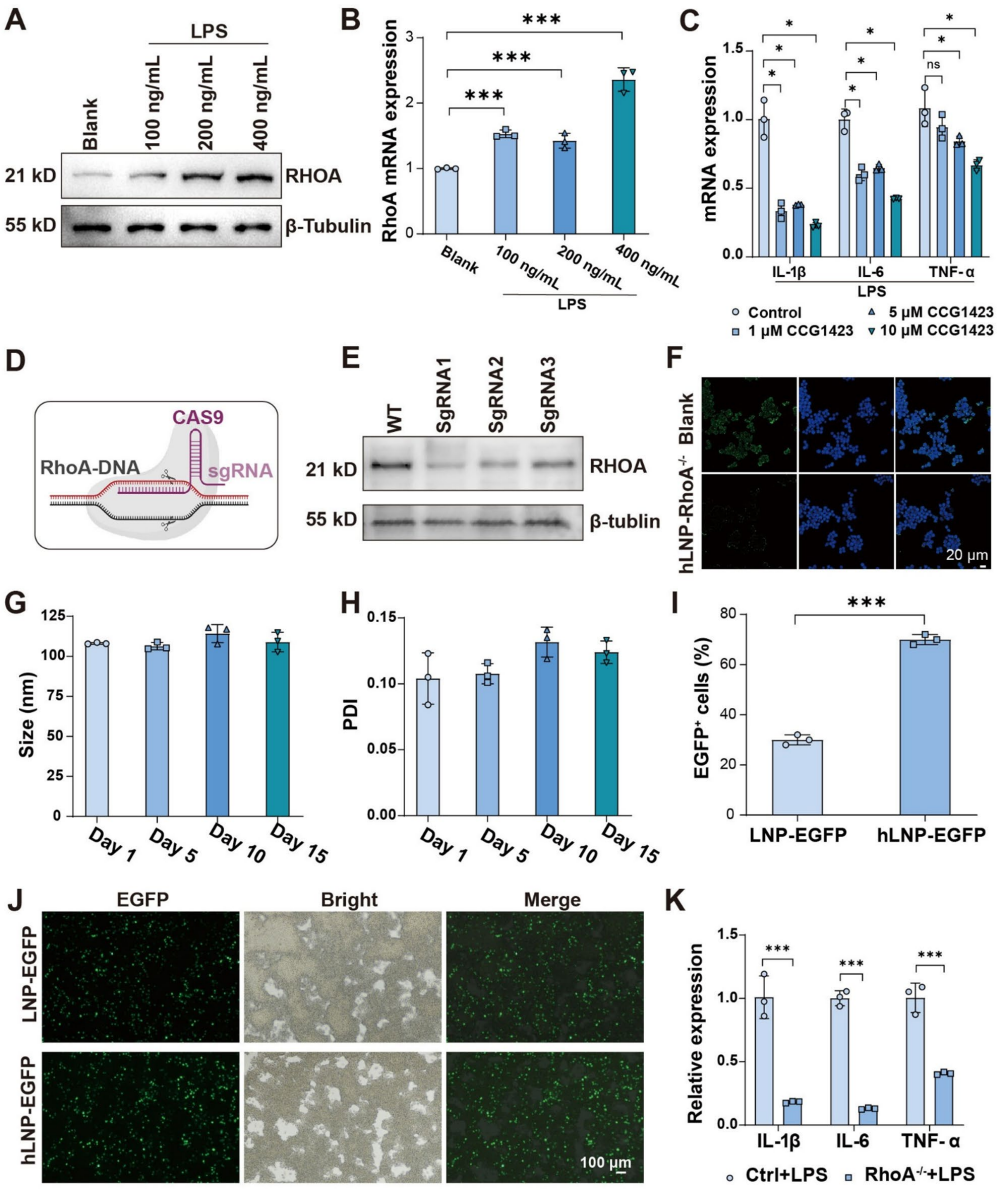
Establishment of the RhoA gene-editing platform and hLNP loading: (**A**) RhoA protein expression in Raw264.7 cells following LPS stimulation. (**B**) RhoA gene expression in Raw264.7 cells following LPS stimulation. (**C**) Levels of inflammatory cytokines after incubation with different concentrations of a RhoA inhibitor in LNP-treated cells. (**D**) Schematic of the RhoA CRISPR/Cas9 editing mechanism. (**E**) Editing efficiencies of three sgRNAs targeting RhoA. (**F**) Immunofluorescence analysis of RhoA expression in Raw264.7 cells. (**G**,** H**) Size and PDI stability of the hLNP-RhoA^−/−^ formulation. (**I**,** J**) Using EGFP mRNA as a model, evaluation of hLNP-mediated mRNA expression in Raw264.7 cells and semi-quantitative analysis. (**K**) Changes in inflammatory cytokine levels in LPS-stimulated Raw264.7 cells following hLNP-RhoA^−/−^ editing. (Data were analyzed by one-way ANOVA and post-hoc multiple comparisons, ns indicates no statistically significant difference, *P < 0.05, **P < 0.01, ***P < 0.001)

Typically, inflammatory phenotype in monocytes/macrophages is induced using LPS as an in vitro inflammation model for various evaluation assays [23]. To validate the robustness of this model, we first assessed changes in RhoA under inflammatory conditions using molecular biology techniques. An LPS concentration gradient was applied at 100, 200, and 400 ng/mL. As shown in Fig. 4A and S3A, the Western blot bands clearly demonstrate a concentration-dependent upregulation of RhoA with increasing LPS levels. We further examined RhoA mRNA expression by qPCR (Fig. 4B). Although there was no significant difference between Raw264.7 cells treated with 100 and 200 ng/mL LPS, the overall trend mirrored the Western blot results. All LPS-treated groups exhibited a significant increase in RhoA mRNA expression, with the 400 ng/mL group showing the most pronounced upregulation. Next, we used qPCR to determine whether inhibiting RhoA facilitates the switch to an anti-inflammatory phenotype [24]. CCG1423 was selected to target the RhoA pathway [25]. As shown in Fig. 4C, the expression of three inflammatory cytokines (IL-1β, IL-6, and TNF-α) exhibited a clear, concentration-dependent decrease in response to CCG1423. From the data, the concentration dependence of CCG1423 was confirmed, indicating that higher doses of the RhoA inhibitor can effectively reduce inflammatory gene expression in macrophages. These results provide preliminary evidence that RhoA inhibition may serve as an effective strategy for suppressing inflammation.

Building on these data, we introduced a CRISPR/Cas9 mRNA/sgRNA system to achieve more precise gene editing of RhoA (Fig. 4D). The hLNP system described above was further used to deliver the CRISPR/Cas9 mRNA/sgRNA system (hLNP-RhoA^−/−^) for RhoA editing. We designed three sgRNA sequences and validated their inhibitory effects on RhoA protein expression by Western blot. As shown in Fig. 4E and S3B, all three sgRNAs suppressed RhoA in LPS-stimulated wild-type cells. We therefore selected sgRNA1 for subsequent experiments. We further evaluated the reduction in RhoA expression by immunofluorescence. As shown in Fig. 4F, RhoA expression in Raw264.7 cells was markedly diminished following RhoA editing (hLNP-RhoA^−/−^). In addition, the 4 °C storage stability of CRISPR/Cas9 mRNA/sgRNA system were detected by the measuring the changes of size and PDI values. As shown in Fig. 4G-H, hLNPs were stable for at least 15 days under 4 °C. To evaluate the transfection efficiency of the hLNP platform in Raw264.7 cells, we used EGFP mRNA as a model cargo. As shown in Fig. 4I and J, the hLNPs achieved a significantly higher transfection efficiency than conventional LNPs. Following RhoA editing, LPS-stimulated RAW264.7 cells exhibited markedly reduced mRNA levels of pro-inflammatory cytokines (IL-1β, IL-6, and TNF-α) (Fig. 4K). These results provide preliminary evidence supporting the effectiveness of RhoA editing.

This hLNP-based RhoA editing primarily targets monocytes/macrophages. To assess the direct impact of this editing on monocyte/macrophage differentiation into osteoclasts, we directly compared TRAP staining in Raw264.7 cells after osteoclast induction following treatment with hLNPs vs. hLNP-RhoA^−/− ^[26]. As shown in Figure S4, RhoA editing resulted in a pronounced reduction in mononuclear cell fusion, in sharp contrast to the control group. This in vitro suppression of osteoclastogenesis following direct editing in Raw264.7 cells indicates a highly pronounced direct effect. Because monocytes/macrophages are our target cells and exert the most direct regulatory effects, we evaluated the pharmacological activity of the empty editing system (without sgRNA) in vitro by measuring inflammatory gene expression *via* qPCR. IL‑1β mRNA levels were used as a readout of the inflammatory phenotype in Raw264.7 cells. As shown in Figure S5, hLNPs carrying sgRNA exhibited the lowest IL‑1β mRNA levels, whereas hLNPs without sgRNA showed no significant anti‑inflammatory effect.

Collectively, these findings substantiate the therapeutic relevance of RhoA in RA, as the CRISPR/Cas9 mRNA-hLNP system enables efficient RhoA editing. Heparinylation markedly increases macrophage transfection efficiency, and EGFP mRNA reporting demonstrates superior protein expression with hLNPs relative to conventional PEGylated LNPs. This enhancement is plausibly attributable to increased macrophage affinity conferred by heparinylation. Following RhoA editing, the pronounced reduction in multiple inflammatory cytokines provides preliminary validation that hLNPs are well-suited for CRISPR/Cas9-mediated gene editing in macrophages.

### *In vitro* anti-inflammation efficacy evaluation of hLNP-RhoA^−/−^

**Fig. 5:**
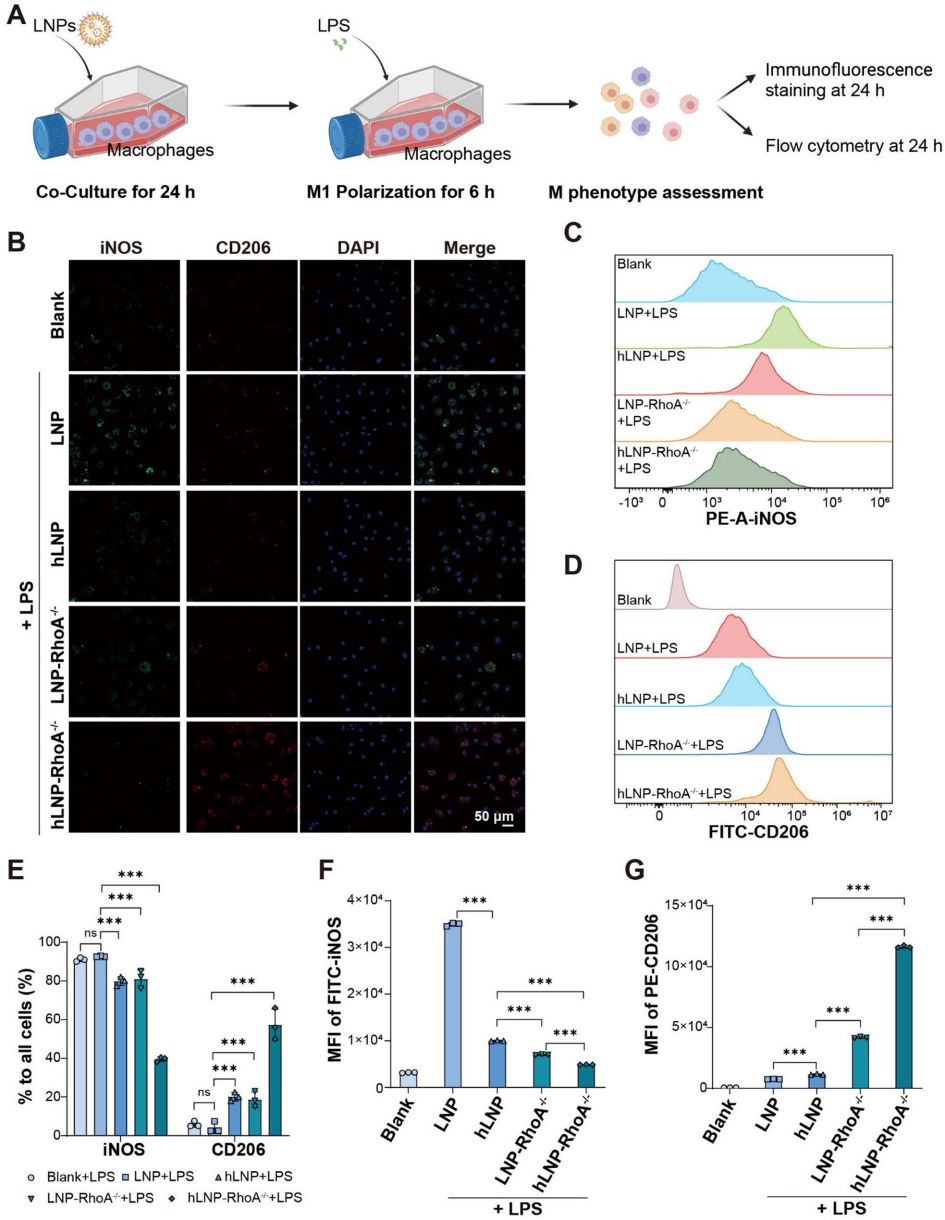
In vitro evaluation of the anti-inflammatory efficacy of hLNP-RhoA^−/−^: (**A**) Schematic of macrophage polarization assessment. (**B**) Polarization of LPS-stimulated Raw264.7 cells after incubation with hLNP-RhoA^−/−^ visualized by immunofluorescent staining of iNOS and CD206 and examined by confocal laser scanning microscopy. (**C–D**) Flow cytometric analysis of iNOS and CD206 immunofluorescent staining to assess polarization in LPS-stimulated Raw264.7 cells after hLNP-RhoA^−/−^ treatment. (**E**) Semi-quantitative analysis of iNOS and CD206 staining by fluorescence microscopy. (**F–G**) Semi-quantitative analysis of iNOS and CD206 staining by flow cytometry. (Data were analyzed by one-way ANOVA and post-hoc multiple comparisons, ns indicates no statistically significant difference, *P < 0.05, **P < 0.01, ***P < 0.001)

Building on the above data, we conducted a preliminary study on the properties of RhoA^−/−^ CRISPR/Cas9 mRNA hLNPs (hLNP-RhoA^−/−^) and evaluated their anti-inflammatory potential in vitro. Macrophages were first edited for RhoA using either conventional LNPs or hLNPs, then stimulated with LPS to induce M1 polarization. Outcomes were assessed by immunofluorescence microscopy and flow cytometry (Fig. 5A). To distinguish polarization states, iNOS and CD206 were used as canonical markers of M1 and M2 macrophages, respectively, quantified with fluorophore-conjugated antibodies [27]. As shown in Fig. 5B, Raw264.7 cells treated with hLNP-RhoA^−/−^ exhibited the strongest CD206 signal and the weakest iNOS signal, indicative of a pronounced M2 bias. Flow cytometry yielded concordant results (Fig. 5C-D).

Quantitative analyses further supported these trends. As shown in Fig. 5E, during immunofluorescence assay, hLNP-RhoA^−/−^ treatment resulted in decreased levels of both iNOS and CD206 compared to hLNP, with a significant difference in the proportion of positive cells (*P* < 0.001), indicating that RhoA editing has a substantial impact on the inflammatory chemotaxis of monocytes. Similarly, LNP-RhoA^−/−^ also showed significant differences compared to LNP (*P* < 0.001), further highlighting the importance of RhoA editing. In addition, the proportion of iNOS-positive cells in the hLNP-RhoA^−/−^ group was only half that of the LNP-RhoA^−/−^ group, while the proportion of CD206-positive cells was more than twice as high. As shown in Fig. 5F-G, significant differences were also observed between the hLNP-RhoA^−/−^ group and all other groups (*P* < 0.001). Collectively, these data indicate that hLNP-RhoA^−/−^ achieves the most effective functional editing and robustly attenuates inflammatory activation.

These data elucidate how hLNP-RhoA^−/−^ modulates macrophage polarization. Building on the hLNP platform, RhoA knockout achieves greater efficacy than conventional LNPs, as suggested by earlier results. Under LPS stimulation, the proportion of M1-polarized Raw264.7 cells after treatment with the hLNP editing system is only half that of conventional LNPs, while the M2-polarized fraction exceeds twice that of the LNP group. This underscores the superior gene-editing performance of hLNPs in macrophages. Moreover, we observed that hLNPs themselves exert significant regulatory effects on polarization: hLNPs loaded with NC mRNA differ markedly from LNPs in M1/M2 outcomes, indicating that the intrinsic immunomodulatory activity of heparinylation should not be overlooked. The immunoregulatory capacity of hLNP-RhoA^−/−^ in macrophages thus arises from the combined effects of heparinylation and RhoA gene editing.

### Preparation of hLNP-RhoA^−/−^@MS

Although hLNP-RhoA^−/−^ has been preliminarily shown to have RhoA editing and anti-inflammatory effects, using LNPs alone is not the optimal solution for intra-articular injection. This is because, following rapid local injection, the sudden high exposure of LNPs to target cells can lead to receptor overload, inhibit cellular uptake, and result in unpredictable loss of the particles [6, 28]. The stability of mRNA therapeutics under physiological conditions remains uncertain. Previous studies have reported that encapsulation techniques, such as hydrogel microspheres, can help maintain the local stability of mRNA LNPs at the disease site, including within the joint cavity [23, 27, 29]. Encapsulation with hydrogels significantly improves the efficiency of gene therapy. In addition to serving as carriers for LNPs, hydrogel microspheres can also enhance lubrication within the joint cavity, thereby reducing inflammatory activation by improving joint mechanical stimulation [27].

**Fig. 6:**
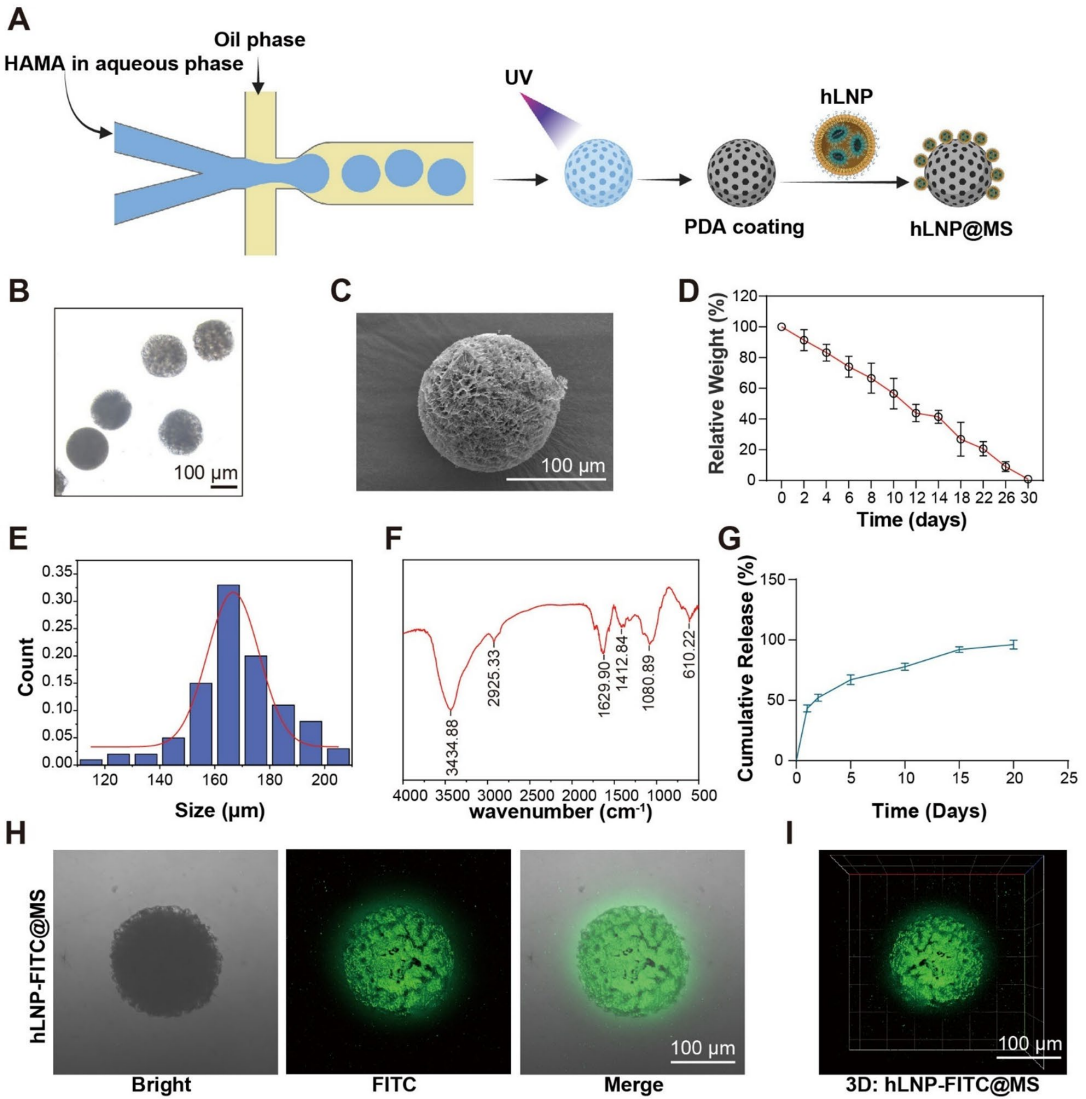
Fabrication, characterization, degradability, and LNP loading of porous HAMA hydrogel microspheres: (**A**) Schematic of microfluidic fabrication of porous HAMA microspheres: droplet generation, freezing with ice-crystal templating, and UV-induced photopolymerization. (**B**) Optical microscopy showing uniform microsphere size and narrow size distribution. (**C**) SEM characterization of the porous internal architecture. (**D**) In vitro degradability of HAMA microspheres in hyaluronidase-containing buffer simulating the joint cavity. (**E**) Size distribution analysis of microspheres prepared by microfluidics. (**F**) FT-IR spectra highlighting characteristic LNP structural peaks. (**G**) Cumulative release profile of FITC-labeled LNPs from PDA-coated microspheres under light-protected conditions. (**H**,** I**) Confocal microscopy confirming LNP loading: FITC fluorescence localized on/within PDA-coated HAMA microspheres

In this study, we employed advanced microfluidic technology to fabricate porous hydrogel microspheres (Fig. 6A). The use of microfluidics allowed for improved control over particle size uniformity and tunability. Methacrylated hyaluronic acid (HAMA) was selected as the base material, and after freezing, gelation was achieved by UV-induced photopolymerization following ice crystal templating [27]. When the ice crystals melted, unique channels were formed, increasing the specific surface area. Under an optical microscope, the uniform particle size of the microspheres was clearly observed (Fig. 6B and E). Their porous structure was further characterized by scanning electron microscopy. Furthermore, under stimulation by hyaluronidase, which is overexpressed in the joint cavity, the HAMA microspheres exhibited significant degradability in simulated in vitro fluid (Fig. 6D).

To achieve efficient physical adsorption of LNPs on the surface of the microspheres, a polydopamine coating was deposited via dopamine self-polymerization in a weakly alkaline solution. This enhanced the interactions, such as hydrogen bonding and van der Waals forces, between the microsphere surface and LNPs, and the porous structure further increased adsorption capacity. Typically, hLNP‑RhoA^‑/‑^@MS was prepared at a ratio of 1 mL of LNP suspension with 50 µg lipids to 1 mg of microspheres. Based on the residual mRNA in the supernatant after Triton X‑100 treatment, the encapsulation efficiency of the composite microspheres exceeded 90%.

In the Fourier-transform infrared spectroscopy (FT-IR) results, The peaks at 3434, 2925, and 1620 cm^− 1^ correspond to hydrogen-bonded O-H/N-H stretching (from HAMA and PDA/water), aliphatic -CH2- stretching (from LNP lipids and other hydrophobic groups), and a band primarily arising from the aromatic conjugation of PDA in the 1610–1625 cm^− 1^ region, potentially overlapping with amide I or carboxylate vibrations, respectively (Fig. 6F). We also labeled the LNPs with FITC by incorporating it into the lipid phase, and monitored the release profile by measuring fluorescence under light-protected conditions (Fig. 6G) [30]. Finally, confocal microscopy revealed FITC fluorescence on the microspheres, confirming the successful loading of LNPs onto the hydrogel microspheres (Fig. 6H and I).

The above results clearly show that hLNPs can be efficiently loaded onto the HAMA microsphere platform. PDA modification enables robust physical adsorption of LNPs and their controlled release, with more than 50% released within 3 days. The use of PDA-modified microspheres or other microcarriers to complex with nanocarriers has also been widely reported in the literature. This broadly applicable strategy has been extensively employed for local delivery, including intra-articular administration.

### *In vitro* cytotoxicity evaluation of hLNP-RhoA^−/−^@MS

**Fig. 7:**
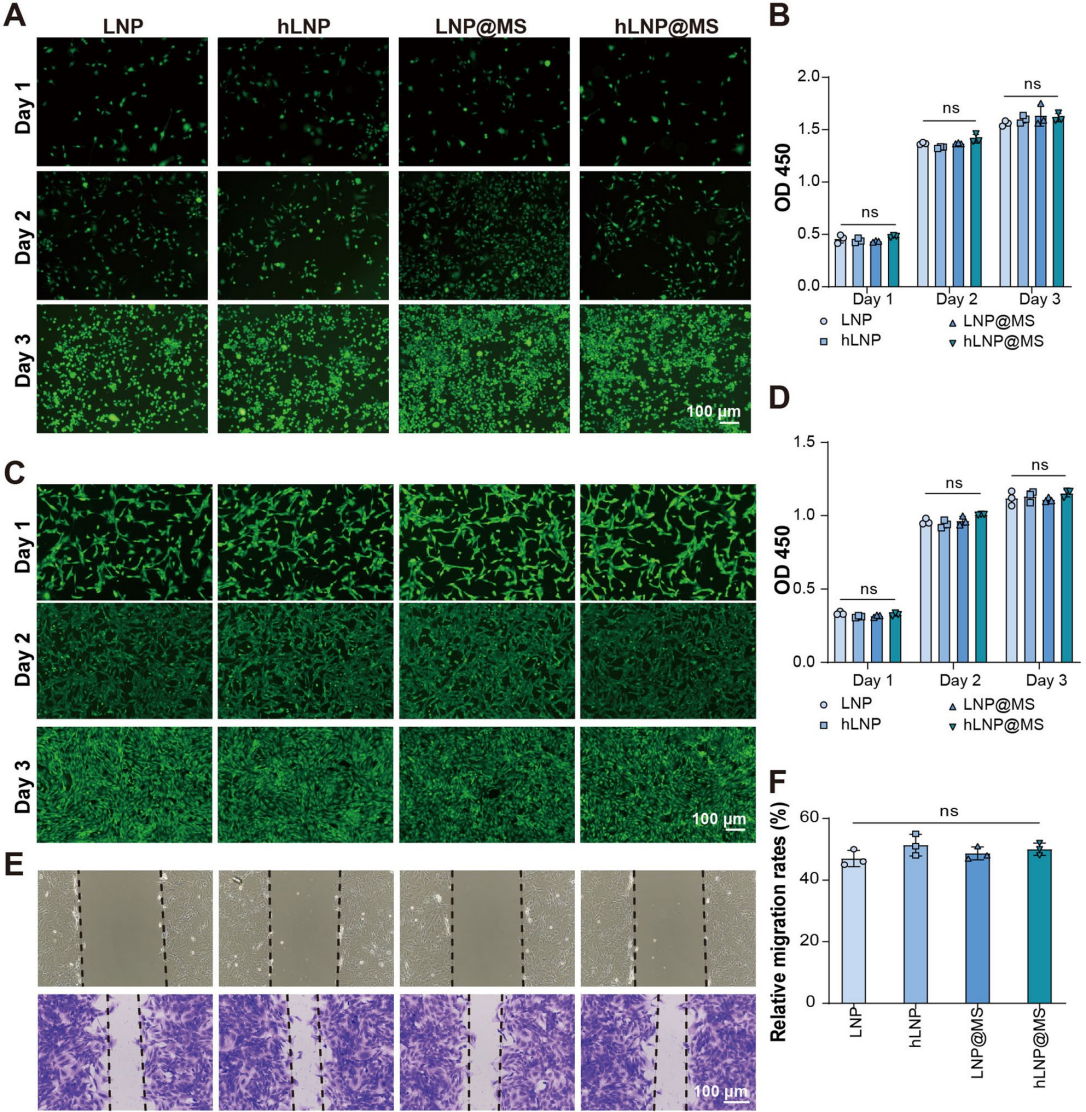
In vitro cytocompatibility and migration assessment of hLNP-RhoA^−/−^@MS: (**A**,** C**) Live/dead staining of Raw264.7 cells (**A**) and BMSCs (**C**) after 1–3 days of co-incubation with LNPs, hLNPs, LNP@MS, or hLNP@MS. Calcein AM (green) marks viable cells; PI (red) marks dead cells. Predominantly viable cells observed in all groups with increasing cell numbers over time. (**B**,** D**) CCK-8 assay of Raw264.7 cells (**B**) and BMSCs (**D**) at days 1, 2, and 3 showing time-dependent increases in cell numbers without significant differences among treatment groups, indicating minimal cytotoxicity. (**E**) Scratch (wound-healing) assay images of BMSCs after 36 h, visualized by crystal violet staining, demonstrating comparable wound closure across groups. (**F**) Semi-quantitative analysis of wound closure showing approximately 50% healing with no significant intergroup differences, suggesting that LNP, hLNP, LNP@MS, and hLNP@MS do not impair BMSC migration. (Data were analyzed by one-way ANOVA and post-hoc multiple comparisons, ns indicates no statistically significant difference, *P < 0.05, **P < 0.01, ***P < 0.001)

Subsequently, we evaluated the cytotoxicity of hLNP-RhoA^−/−^@MS using live/dead staining, a CCK-8 assay, and a scratch (wound-healing) assay. First, Raw264.7 and BMSCs were co-incubated with LNPs and microspheres for 1 ~ 3 days, and cell viability was assessed using Calcein AM/PI double staining, where live cells emit green fluorescence and dead cells emit red fluorescence [31]. As shown in Fig. 7A and C, nearly all groups were predominantly composed of viable cells, and cell numbers increased noticeably with longer incubation times.

To enable quantitative analysis, we performed a CCK-8 assay. After 1, 2, and 3 days of co-incubation, Raw264.7 and BMSCs in each well were treated with CCK-8 working solution and measured using a microplate reader. As shown in Fig. 7B and D, there was a clear increase in cell numbers over time, with no significant differences among the groups. Together with the live/dead staining, these results indicate that LNP, hLNP, LNP@MS, and hLNP@MS exhibit no obvious cytotoxicity, nor are there significant cytotoxicity differences among them.

We evaluated treatment effects on cell migration using a scratch wound-healing assay. To minimize proliferation, cells were maintained in serum-free medium. For BMSCs, the scratch area was visualized after 36 h of incubation by crystal violet staining to better observe wound closure. As shown in Fig. 7E, all groups exhibited comparable wound closure. Semi-quantitative analysis (Fig. 7F) indicated an average healing rate of approximately 50% with no significant differences among groups. These findings suggest that none of the treatments materially affect BMSC migratory capacity, further supporting their cellular safety profile.

It is evident that hLNP-RhoA^−/−^@MS exhibits no appreciable cytotoxicity and does not impair BMSC migration. Conventional LNPs are highly safe nucleic acid delivery systems with extensive clinical use and well-established safety profiles [6]. Likewise, HAMA microspheres and PDA coatings are widely validated as biocompatible biomaterials [32]. Here, we provide the first verification of the safety of LNP heparinylation, akin to PEGylation, heparinylation represents a highly biocompatible LNP surface modification.

### *In vitro* RNA-sequencing analysis of hLNP-RhoA^−/−^@MS

**Fig. 8:**
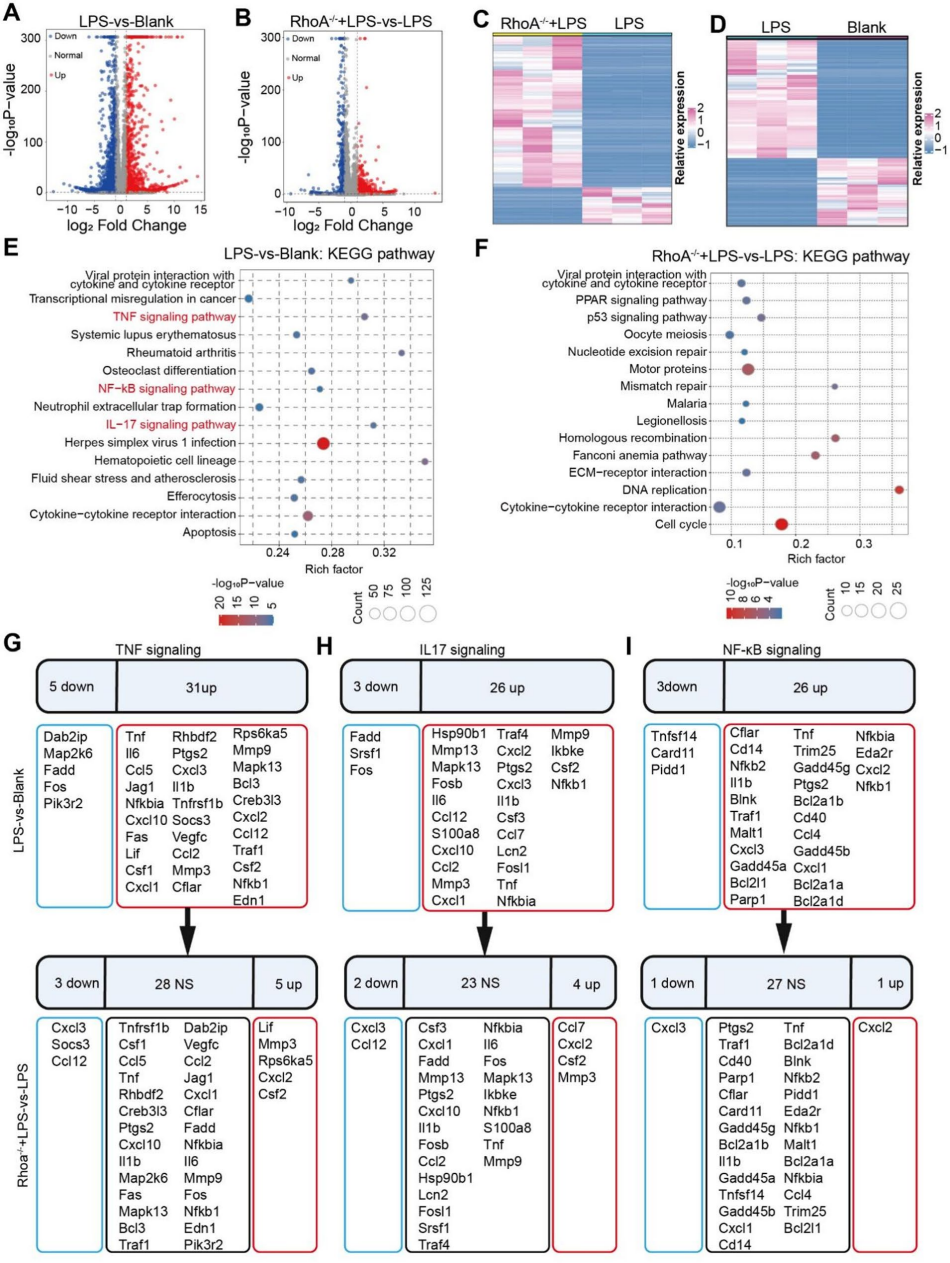
RhoA deficiency suppresses LPS-induced activation of inflammation-related signaling pathways in macrophages: (**A**,** B**) Volcano plots illustrating differentially expressed genes for LPS vs. Blank (**A**) and RhoA^⁻/⁻^ + LPS vs. LPS (**B**); (**C**,** D**) Heatmaps showing gene expression profiles across treatment groups; (**E**,** F**) KEGG pathway enrichment analyses. LPS predominantly activates the TNF, NF-κB, and IL-17 signaling pathways, whereas Rhoa deletion markedly attenuates their enrichment; (**G–I**) Expression of genes within the TNF-α (**G**), IL-17 (**H**), and NF-κB (**I**) pathways. Red denotes upregulated genes, blue denotes downregulated genes, and black indicates no significant change (NS). These data indicate that RhoA deficiency dampens LPS-driven activation of core inflammatory pathways

To delineate the role of RhoA in inflammatory responses, we performed transcriptomic profiling of macrophages stimulated with LPS. The results showed that LPS treatment induced widespread gene upregulation (Fig. 8A and C). In contrast, RhoA deficiency markedly reduced the number of LPS-induced differentially expressed genes (Fig. 8B and D).

KEGG analysis indicated that LPS primarily activates inflammation-related pathways, including TNF, NF-κB, and IL-17 signaling (Fig. 8E). Notably, loss of RhoA significantly attenuated the enrichment of these pathways, shifting the transcriptomic focus toward fundamental processes such as DNA repair and the cell cycle (Fig. 8F). Further analysis revealed that LPS robustly upregulated core genes in the TNF pathway (Tnf, Il6, Il1b, Nfkbia; Fig. 8G), the IL-17 pathway (Cxcl1, Ccl2, Csf2; Fig. 8H), and the NF-κB pathway (Tnf, Ccl4, Cd40, Nfkbia; Fig. 8I), whereas RhoA deficiency markedly suppressed the expression of these inflammatory hub genes.

GO enrichment analysis corroborated these findings. Under LPS stimulation, differentially expressed genes were significantly enriched in processes such as immune cell activation, cytokine production, and cell chemotaxis, as well as functions like cytokine receptor binding and chemokine activity (Figure S6A). By contrast, in the absence of RhoA, GO enrichment shifted toward mitosis, chromosome segregation, DNA replication, and microtubule-related functions (Figure S6B), suggesting weakened inflammatory signaling and a redistribution of transcriptional resources to cell-cycle and DNA metabolic programs.

While the RNA-seq data provide preliminary evidence that pathways such as TNF and NF-κB pathways are suppressed, the direct downstream signaling pathways governing macrophage polarization and inflammatory responses following RhoA editing remain unclear. Therefore, Western blot analysis was applied to examine changes in the phosphorylation levels of key signaling proteins, thereby corroborating the mechanism of inflammation suppression after RhoA editing. As shown in Figure S7, treatment with hLNP‑RhoA (hLNP‑RhoA^−/−^) reduced P65 phosphorylation (p-P65) in Raw264.7 cells following LPS stimulation, indicating inhibition of the NF‑κB pathway. This result corroborates the RNA‑seq findings, which suggested decreased NF‑κB pathway activity after RhoA editing.

In summary, RhoA plays an important role in LPS-driven inflammation. Its loss substantially dampens activation signaling pathways, especially NF-κB pathway, and reduces the expression of cytokines and chemokines, suggesting that RhoA can serve as a target gene to validate the gene-editing functionality of the hLNP platform.

### *In vivo* anti-RA efficacy evaluation of hLNP-RhoA^−/−^@MS

**Fig. 9:**
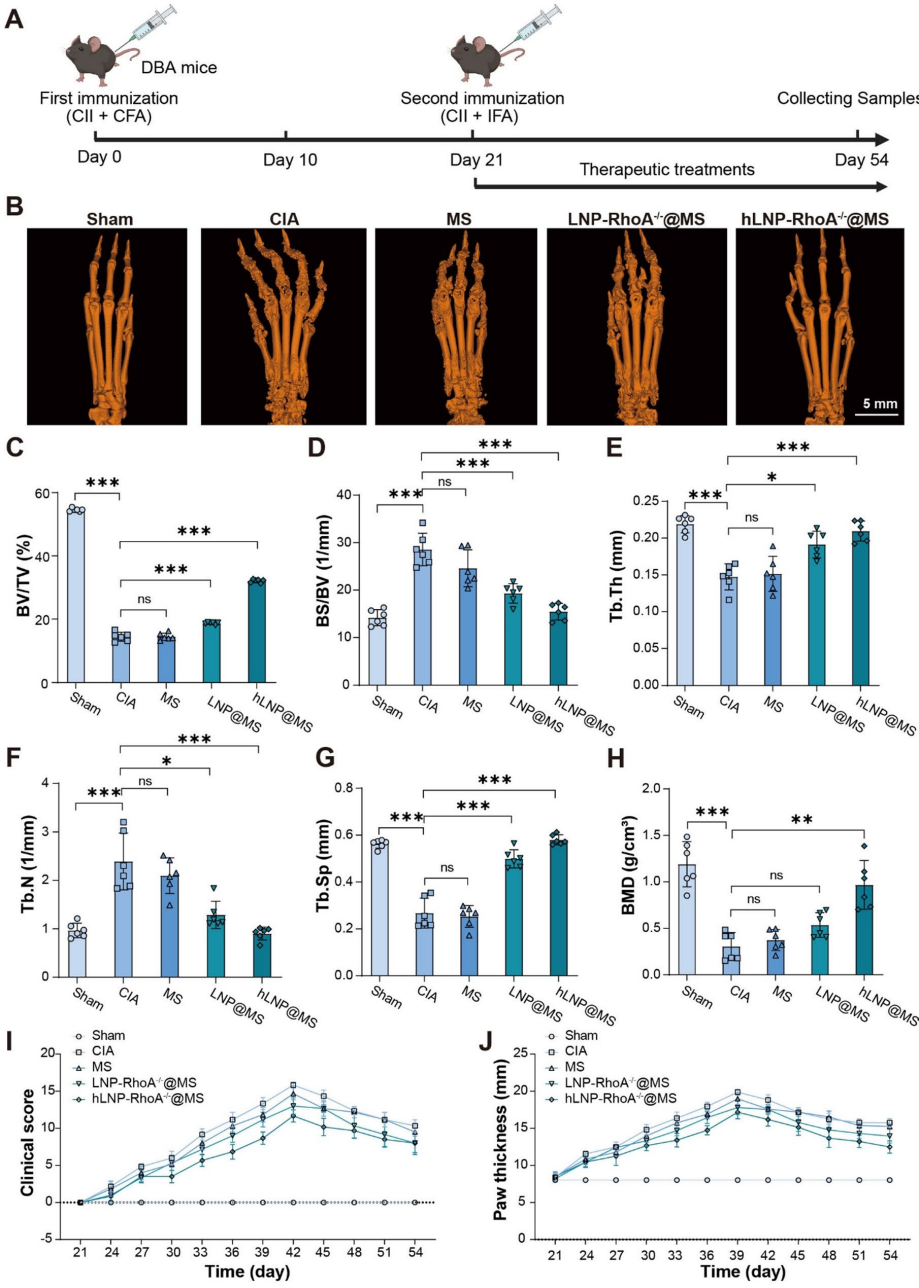
In vivo anti-RA efficacy of hLNP-RhoA^−/−^@MS in the CIA mouse model: (**A**) Schematic of the murine collagen-induced arthritis (CIA) model and treatment regimen. (**B**) Representative Micro-CT 3D reconstructions of whole-paw skeletons showing that hLNP-RhoA^−/−^@MS markedly suppresses bone erosion, closely resembling the Sham group; other CIA groups display varying degrees of erosion. (**C–H**) Quantitative Micro-CT analyses of bone parameters: BV/TV, BS/BV, Tb.Th, Tb.N, Tb.Sp, and BMD. (**I**) Clinical arthritis scores over time, where lower scores indicate milder disease. (**J**) Paw thickness measurements reflecting joint swelling. (Data were analyzed by one-way ANOVA and post-hoc multiple comparisons, ns indicates no statistically significant difference, *P < 0.05, **P < 0.01, ***P < 0.001)

We selected the murine collagen-induced arthritis (CIA) model to evaluate the anti-RA efficacy of hLNP-RhoA^−/−^@MS (Fig. 9A) [33]. Compared with models such as adjuvant-induced arthritis (AIA), the CIA model offers clear advantages in recapitulating the pathogenesis of RA [34, 35].

Here, RA is characterized by pronounced bone erosion. We evaluated bone erosion in CIA mice across groups using Micro-CT. After excising the mouse paws, we performed Micro-CT scanning and conducted bone tissue parameter analyses on the entire paw skeleton. The Micro-CT-reconstructed paw bone structures clearly show that hLNP-RhoA^−/−^@MS suppresses bone erosion. In contrast, the other three CIA groups exhibit evident bone erosion to varying degrees. The hLNP-RhoA^−/−^@MS group most closely resembles the Sham group (Fig. 9B). We further analyzed a series of bone parameters based on Micro-CT 3D reconstructions, including bone volume to total volume (BV/TV), bone surface to bone volume (BS/BV), trabecular thickness (Tb.Th), trabecular number (Tb.N), trabecular separation (Tb.Sp), and bone mineral density (BMD) [36, 37]. As shown in Fig. 9C-H, all bone parameters indicate markedly reduced bone erosion in the hLNP@MS group, with values closest to those of the Sham group. Compared with the CIA model group, the hLNP@MS group exhibited significant improvements across every parameter (*P* < 0.001). Notably, in BV/TV, Tb.Sp, and BMD, the hLNP@MS group showed at least a twofold increase compared with the model group.

We further evaluated the clinical scores of each group and measured paw thickness to additionally assess the remission of RA. A higher clinical score indicates more severe RA symptoms. Because the degree of swelling can vary among the joints of four limbs, our scoring system accounts for all four limbs, with a total score of 20 points. As shown in Fig. 9I, the hLNP@MS group consistently maintained the lowest scores among all treatment groups, and by the end of treatment its score was comparable to that of the LNP@MS group. In contrast, the MS group showed minimal therapeutic benefit and remained closest to the CIA model group. Paw thickness provides a more direct readout of joint swelling. As shown in Fig. 9J, the hLNP@MS group also exhibited the lowest paw thickness throughout, with a rapid decline after reaching a predefined endpoint at week 39. Ultimately, the CIA group showed the most pronounced swelling, followed by the hLNP@MS group, whereas the MS group closely resembled the CIA model group. This pattern is consistent with the trends observed in the Micro-CT and clinical score data.

Unexpectedly, hLNP-RhoA^−/−^@MS displayed obvious suppression of RA-related bone erosion, rendering the bone status of CIA mice nearly comparable to that of healthy controls. This outcome clearly stems from highly effective macrophage-targeted intervention with dual mechanisms: heparinylation itself exerts potent anti-inflammatory effects, and it also enhances the delivery of CRISPR/Cas9 mRNA/sgRNA to macrophages by hLNPs. The combination of these effects produces superior therapeutic efficacy. In addition, MS shows a modest capacity to ameliorate RA, though its difference from the model group is not statistically significant. Nevertheless, microsphere carriers have become a classic tool for intra-articular injection in RA and are widely recognized for their therapeutic utility. Accordingly, all three treatment groups in this study were designed around the microsphere platform. Ultimately, these data reaffirm the efficacy of hLNP-RhoA^−/−^@MS.

### *In vivo* histopathological evaluation of hLNP-RhoA^−/−^@MS

After euthanizing the CIA mice, we collected sagittal sections of the ankle joints for histopathological analysis. We focused on pathological changes in the medial tubercle of the talus and the tubercle of the navicular [38]. This is because these regions combine several advantages, stress concentration, high sensitivity to synovial invasion, reliable anatomical localization, and good quantifiability, making them ideal windows for assessing RA/CIA-related pathological changes in the ankle. We performed multiple histopathological analyses, including TRAP staining, H&E staining, and Safranin O staining.

**Fig. 10:**
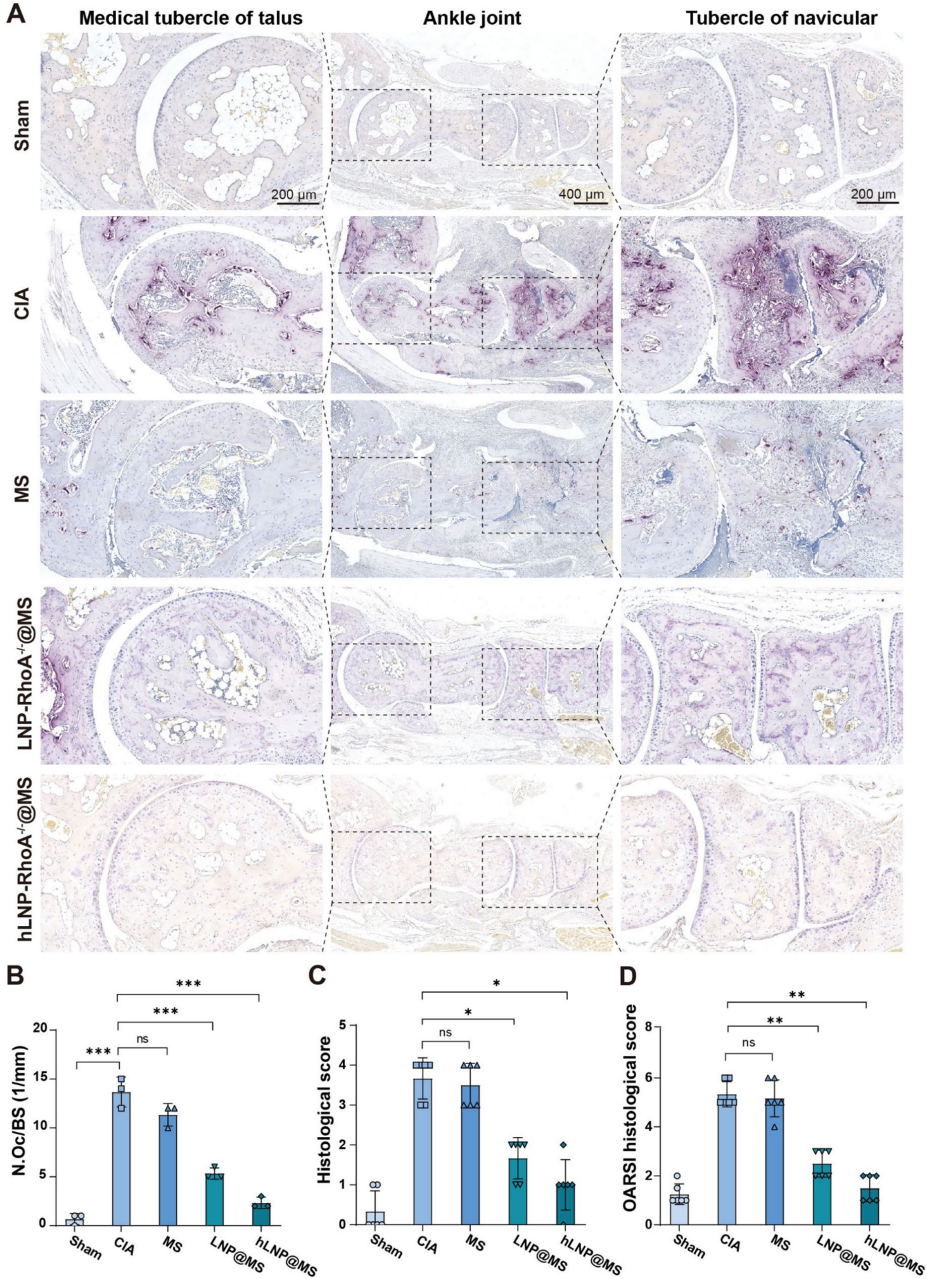
Histopathological evaluation of osteoclast activity and joint pathology: (**A**) TRAP staining of paw sections showing osteoclasts (red/magenta) along bone surfaces. (**B**) Quantification of osteoclasts by N.Oc/BS. (**C**) Histological scores from H&E-stained sections. (**D**) OARSI scores from Safranin O-stained sections. (Data were analyzed by one-way ANOVA and post-hoc multiple comparisons, ns indicates no statistically significant difference, *P < 0.05, **P < 0.01, ***P < 0.001)

TRAP staining is used to identify and quantify osteoclasts and to evaluate bone resorption activity. As shown in Fig. 10A, the red/magenta staining clearly demonstrates the presence and distribution of osteoclasts along the bone surface. The hLNP@MS group shows the weakest staining, closely resembling the Sham group, whereas the MS and LNP@MS groups still exhibit varying degrees of staining. We further analyzed the histological sections using the Number of Osteoclasts per Bone Surface (N.Oc/BS) metric. As shown in Fig. 10B, the hLNP@MS group exhibits a marked reduction in N.Oc/BS compared with the CIA group (*P* < 0.001). Similarly, the LNP@MS group shows a significant decrease versus the CIA group (*P* < 0.001), though to a lesser extent than hLNP@MS. In contrast, although the MS group shows a numerical reduction in N.Oc/BS, this difference is not statistically significant compared with the CIA group (*P* > 0.05).

Subsequently, we conducted further pathological analyses of the H&E (Figure S8) and Safranin O staining (Figure S9) images and quantified the data as histological and OARSI histological scores, respectively. As shown in Fig. 10C, the hLNP@MS group achieved scores most comparable to the Sham group and differed significantly from the CIA group (*P* < 0.05). The LNP@MS group had slightly higher scores than hLNP@MS but still showed a significant difference versus the CIA group (*P* < 0.05). In contrast, the MS group did not differ significantly from the CIA group. Finally, quantitative analysis of the Safranin O staining (Fig. 10D) yielded similar results, with one distinction: the differences between the hLNP@MS and LNP@MS groups vs. the CIA group were even more significant (*P* < 0.01). Across all three pathological metrics, both gene-editing groups (hLNP@MS and LNP@MS) showed a significant attenuation of RA pathology compared with the CIA group, with hLNP@MS demonstrating the most pronounced therapeutic effect.

Through histopathological evaluation, the therapeutic effects of hLNP-RhoA^−/−^@MS on RA joints were further validated. TRAP staining revealed robust inhibition of osteoclastogenesis, which aligns well with the Micro-CT findings of reduced bone erosion. Given that osteoclasts derive directly from the monocyte/macrophage lineage, we infer that RhoA editing in macrophages is the primary driver of the observed suppression of osteoclast activity. Moreover, joint inflammation was markedly alleviated, and decreased paw thickness reflected reduced joint swelling. Collectively, these data underscore the outstanding therapeutic potential of hLNP-RhoA^−/−^@MS for RA.

This study demonstrates that a heparinized mRNA LNP microsphere platform delivering CRISPR targeting RhoA effectively alleviates RA pathology in the CIA model. These findings are consistent with RNA sequencing results and other foundational studies on RhoA. In general, RhoA directly regulates cytoskeletal dynamics, NF-κB signaling, and osteoclast activity, processes closely linked to inflammation and bone erosion. In our system, heparin functionalization may reshape the nanoparticle protein corona and modulate receptor engagement within the inflammatory microenvironment, thereby enhancing macrophage targeting and uptake compared with PEGylated LNPs. In vivo, we observed improved bone parameters and reduced clinical severity, directly confirming that macrophage editing influences not only inflammatory progression but also the balance between osteogenesis and bone resorption. Overall, these results support heparinized LNPs as a macrophage focused gene editing platform for RA with translational potential.

Departing from prior approaches, this work advances LNP delivery by substituting PEGylation with heparinylation, thereby better adapting the platform for local, in situ macrophage targeting. In parallel, we identify macrophage RhoA as a promising therapeutic target in RA, demonstrating its regulatory influence across multiple related pathways. Using this macrophage-adapted mRNA LNP strategy, we achieved localized RhoA gene editing within RA joints. Convergent in vitro and in vivo data substantiate our hypothesis, establishing a paradigm for local gene therapy in RA that couples an enhanced delivery vehicle with a novel target.

## Conclusion

In summary, this work reports a heparin-modified, intra-articularly injectable macrophage-targeted CRISPR/Cas9 gene-editing LNP microcomplex (hLNP-RhoA^−/−^@MS) that enables precise knockout of the bioinformatically identified target RhoA, ultimately achieving effective treatment of RA. Replacing PEG with heparin for LNP surface modification resulted in higher transfection efficiency of the delivered mRNA/sgRNA. Moreover, incorporation of the microsphere system markedly enhanced the stability and intra-articular retention of the LNPs. In vitro RNA-seq analyses revealed broad suppression of multiple key inflammation-related signaling pathways following RhoA editing. In the in vivo CIA mouse model, hLNP-RhoA^−/−^@MS produced pronounced alleviation of joint inflammation, inhibition of cartilage and bone damage, and remodeling of the immune microenvironment, confirming its robust anti-RA efficacy. Therefore, this study provides a feasible and highly effective gene-editing strategy for local RA therapy.

## Experimental section

### Bioinformatics analysis of clinic RNA-seq dataset of RA patients

Public RNA-seq data for rheumatoid arthritis (RA) were obtained from GEO (GSE97779), including RA patient samples and healthy controls. When raw FASTQ files were available, reads were quality-checked (FastQC), trimmed (Trim Galore), aligned to GRCh38 using STAR, and summarized to gene counts with featureCounts (GENCODE annotation); otherwise, provided count matrices were used directly. Differential expression analysis was performed in R using DESeq2 with default normalization and a negative binomial GLM (Wald test), applying Benjamini–Hochberg correction (significance thresholds: |log2FC| ≥ 1, FDR < 0.05). Volcano plots and box/violin plots were generated with ggplot2; TNF, IL6, and IL1B expression was compared between groups using normalized counts (vst) and Wilcoxon tests. Upregulated DEGs were subjected to GO (BP, MF) and KEGG enrichment using clusterProfiler (org.Hs.eg.db; BH-adjusted q < 0.05). Disease association enrichment was performed against DisGeNET and visualized in Cytoscape. Gene intersections across small GTPase signaling, monocyte differentiation, and lymphocyte differentiation were identified (VennDiagram), highlighting RHOA. All analyses used scripted workflows with fixed seeds, and software versions/parameters were recorded for reproducibility.

### Preparation of hLNP-RhoA^−/−^

hLNPs were formulated by replacing the PEG-lipid component in a conventional four-lipid LNP system with low-molecular-weight heparin-cholesterol (LMWH-cholesterol) at the same molar fraction (1.5%) [39]. Briefly, the lipid phase (ionizable lipid, helper phospholipid, cholesterol, and LMWH-cholesterol or PEG-lipid for controls, molar ratio of 50%: 1.5%: 10%: 38.5%) was dissolved in ethanol, and the aqueous phase contained mRNA (RhoA CRISPR components or model mRNA) in 25 mM sodium acetate buffer (pH 4.0). Using a microfluidic mixer (Unigen Biotech Co., Suzhou, China), the aqueous and lipid streams were combined at a fixed flow rate ratio (3:1, aqueous: ethanol) and total flow rate of 8 ~ 12 mL/min, followed by immediate dilution in PBS, centrifugal diafiltration to remove ethanol and free mRNA, and concentration to the desired dose. The same device and parameters were used for both LNPs and hLNPs to ensure comparability.

Particle size and polydispersity index (PDI) were measured by dynamic light scattering (DLS), and morphology was examined by transmission electron microscopy (TEM). Encapsulation efficiency was quantified by RiboGreen with and without detergent lysis. For cellular trafficking, Cy5.5-labeled mRNA LNPs were incubated with Raw264.7 cells for 1 and 4 h, lysosomes were stained with LysoTracker Green, and colocalization was imaged by confocal microscopy. Where applicable, FT-IR spectra of related materials (microspheres with LNPs) were recorded to confirm characteristic bands. All measurements were performed on matched LNP and hLNP formulations prepared under identical conditions.

### Preparation of hLNP-RhoA^−/−^@MS

Methacrylated hyaluronic acid (HAMA) was prepared by reacting sodium hyaluronate with methacrylic anhydride under alkaline conditions, followed by dialysis and lyophilization [40]. HAMA precursor solution (1 ~ 2% w/v in PBS) containing photoinitiator (0.1% w/v LAP) was used as the dispersed phase in a flow-focusing microfluidic device, with fluorinated oil/surfactant as the continuous phase to generate monodisperse droplets. Collected droplets were snap-frozen (−20 to −80 °C) to induce ice-crystal templating and then photopolymerized under UV light (365–405 nm) while maintained in the frozen state. After thawing to melt ice and create interconnected channels, the emulsion was broken with perfluorinated solvent, and microspheres were extensively washed with PBS and sieved to narrow the size distribution. To promote efficient physical adsorption of lipid nanoparticles, microspheres were coated with polydopamine by incubating in freshly prepared dopamine solution (2 mg/mL in 10 mM Tris-HCl, pH 8.5) for 2 ~ 4 h at room temperature with gentle agitation, followed by thorough PBS washing. LNPs or hLNPs (optionally FITC-labeled via incorporation into the lipid phase) were incubated with PDA-coated microspheres in PBS for 1 ~ 2 h to allow adsorption; unbound particles were removed by washing.

We prepared the hLNP-microsphere composite (hLNP-RhoA^−/−^@MS) at a 1 mL of LNP suspension with 50 µg/mL lipids to 1 mg microsphere ratio and quantified adsorption by OD260 after Triton X‑100 disruption. Briefly, 1 mg HAMA‑PDA microspheres were mixed with the LNP‑mRNA suspension and incubated at 4 °C with gentle rocking for 1 h, then centrifuged at 2000 rpm for 5 min to collect the supernatant; Triton X‑100 was added to 0.1% v/v and incubated for 10 min at room temperature to release encapsulated mRNA, followed by OD260 measurement at 260 nm (NanoDrop) to determine unadsorbed mRNA. Based on the measurements, the actual encapsulation efficiency of mRNA in the composite microspheres exceeded 90%.

For degradability assessment, microspheres were incubated with hyaluronidase in simulated synovial fluid or PBS at 37 °C and monitored for mass loss and morphology changes. Loading efficiency and release profiles were quantified by fluorescence of FITC-LNPs under light-protected conditions at 37 °C, sampling supernatants over time. Structural features were examined by optical microscopy for size uniformity and SEM for porosity; surface chemistry was characterized by FT-IR. Confocal microscopy was used to visualize FITC signals on or within microspheres, confirming successful LNP loading.

### *In vitro* cytotoxicity analysis

Cells were harvested by trypsinization, resuspended in complete medium, and seeded into 24-well plates at a density of 2.0 × 10^4^ cells per well [41]. After attachment, cells were exposed to the indicated treatment conditions, and viability was assessed at 1, 4, and 7 days using the Cell Counting Kit‑8 (CCK‑8; Beyotime, Shanghai, China). At each time point, 200 µL CCK‑8 working solution was added to each well and incubated for 2 h at 37 °C in the dark, after which absorbance at 450 nm was recorded using a microplate reader. Results are summarized in the accompanying table.

### *In vitro* live/dead staining

Cells were plated in 24‑well plates and treated as planned. For adherent cells, the culture medium was removed and, if needed, cells were rinsed once with PBS to eliminate residual serum [15]. A Calcein‑AM/PI working solution (250 µL per well) was added, followed by a 10‑min, light‑protected incubation at 37 °C. Fluorescence microscopy was used to assess viability: live cells appeared green (Calcein-AM, Ex/Em 494/517 nm) and dead cells appeared red (PI, Ex/Em 535/617 nm).

### Macrophage polarization

Macrophage polarization was induced with slight adjustments to established protocols. Raw264.7 cells (cultured in DMEM with 10% FBS) were plated in 6‑well plates at 2 × 10^5^ cells per well and allowed to adhere overnight [42]. Cells were then exposed to LPS (100 ng/mL) in the presence of LNPs for 24 h. Polarization status was assessed by flow cytometry using iNOS as an M1 marker and CD206 as an M2 marker. All assays were conducted with at least three independent biological replicates.

### qRT-PCR analysis

Total RNA was isolated with FreeZol Reagent (Vazyme, China) following the manufacturer’s protocol. Complementary DNA was generated using HiScript III RT SuperMix for qPCR with gDNA wiper (Vazyme, China). qRT‑PCR was carried out with ChamQ Universal SYBR qPCR Master Mix (Vazyme, China) on a real-time thermal cycler. Gene expression levels were normalized to GAPDH, and relative quantification was determined by the 2^−ΔΔCT^ method. Each gene was analyzed with at least three independent biological replicates.

### Immunofluorescence staining analysis

Cells were fixed with 4% paraformaldehyde for 15 min, then blocked for 30 min using QuickBlock™ Blocking Buffer (Beyotime, China). Samples were incubated overnight at 4 °C with primary antibodies against RhoA (Proteintech, 1:50). The following day, after three PBS washes, cells were incubated for 1 h at room temperature with Alexa Fluor 488-conjugated secondary antibodies (goat anti‑rabbit IgG H&L or goat anti‑mouse IgG H&L; Beyotime, China). Actin‑Tracker Red-555 (Beyotime, China) was then applied for 1 h, and nuclei were counterstained with DAPI (Beyotime, China). Images were captured on a laser confocal microscope, and mean fluorescence intensity or the number of positive cells per group was quantified using ImageJ.

### Western blot analysis

Cells were lysed in RIPA buffer, and total protein was quantified using a BCA assay (Beyotime, China). Equal amounts of protein were separated by SDS‑PAGE (Beyotime, China) and transferred onto PVDF membranes (Beyotime, China). Membranes were blocked with QuickBlock™ Blocking Buffer (Beyotime, China) for 30 min, then incubated overnight at 4 °C with primary antibodies against RhoA (Proteintech, 1:1000) and β‑tubulin (Proteintech, 1:1000). After three washes with Western Wash Buffer (Beyotime, China), membranes were incubated with HRP-conjugated secondary antibodies, goat anti‑mouse IgG (H + L) or goat anti‑rabbit IgG (H + L) (Proteintech, China), for 1.5 h at room temperature. Immunoreactive bands were detected using an enhanced chemiluminescence substrate (NcmECL Ultra; New Cell & Molecular Biotech, China).

### RNA sequencing

Total RNA was isolated using the RNeasy kit (Vazyme) following the manufacturer’s protocol. RNA integrity was evaluated on an Agilent 4200 TapeStation (G2991A, Agilent, CA), and concentrations were determined with a Qubit 3.0 Fluorometer (Q33216, Thermo Fisher Scientific) and/or a NanoDrop One spectrophotometer (Thermo Fisher Scientific). Libraries were prepared from qualified RNA using the VAHTS Stranded mRNA‑seq Library Prep Kit (NR602, Vazyme, China) according to the provided instructions, and library quality was verified by Qubit and TapeStation. Sequencing was performed on an Illumina platform using paired‑end 150 bp (PE150) reads. Sequencing‑by‑synthesis chemistry was employed, wherein DNA clusters formed on the flow cell are extended with fluorescently labeled dNTPs by DNA polymerase; the emitted fluorescence upon nucleotide incorporation is imaged each cycle and converted into base calls and FASTQ reads by instrument software. Downstream analyses were conducted in a Linux environment with R/RStudio used for statistical analysis and visualization of the transcriptomic data.

### *In vivo* RA modelling and hLNP-RhoA^−/−^@MS treatment

First, thirty 8 ~ 9‑week‑old DBA/1 mice were purchased from Beijing Vital River Laboratory Animal Technology Co., Ltd. and acclimated for 1 week in the SPF animal facility. The mice were then randomly assigned to three groups: Sham, collagen‑induced arthritis (CIA), CIA plus MS intervention (MS), CIA plus LNP-RhoA^−/−^@MS (LNP-RhoA^−/−^@MS) and CIA plus hLNP-RhoA^−/−^@MS (hLNP-RhoA^−/−^@MS) with 6 mice per group. The CIA model was established by two rounds of immunization. A water‑in‑oil emulsion was prepared by mixing complete Freund’s adjuvant (CFA, 4 mg/mL) with type II collagen solution (2 mg/mL) at a 1:1 volume ratio, and 100 µL of the emulsion was injected subcutaneously at the distal tail of mice in the CIA and CIA + MT groups [43]. The Sham group received an equal volume of normal saline. A booster immunization was administered 21 days after the first injection. For the second immunization, an emulsion of type II collagen solution with incomplete Freund’s adjuvant (same 1:1 ratio) was injected subcutaneously at the base of the tail. The Sham group again received an equal volume of saline. After the second immunization, injections were made from the plantar or palmar side toward the periarticular soft tissues of the ankle or wrist. The two experimental groups received orthotopic intra-articular injections into the joints of all four limbs once per week, administering LNP-RhoA^−/−^@MS and hLNP-RhoA^−/−^@MS, respectively (10 µL per side, containing LNP with 40 µg/mL lipid concerntration). The MS group received an equal volume of microspheres. All groups were euthanized on day 54 after the initial immunization. Specimens were collected and processed by decalcification followed by paraffin embedding.

Starting 21 days after the second immunization, arthritis scores were recorded every 3 days from day 24 to day 54 after the first immunization. A practical CIA mouse clinical scoring system uses 0 ~ 5 points per limb (maximum 20 per animal), assessing erythema, swelling extent, and functional impact, and assigning the highest applicable category per limb. Definitions: 0 = normal; 1 = mild redness/swelling in a single toe/finger joint; 2 = ≥ 2 toe/finger joints involved or mild ankle/wrist involvement; 3 = moderate ankle/wrist swelling or both distal and proximal joints involved with weight-bearing preserved; 4 = marked redness, heat, deformity, and limping; 5 = diffuse paw/hand swelling with deformity or near non–weight-bearing. Begin blinded scoring at a consistent time daily or every other day from day 14 post-induction, sum the four limb scores (max 20), and optionally track body weight and paw thickness. If a limb approaches 5 points or the total score ≥ 16 with evident distress, provide analgesia or humanely terminate per ethical guidelines.

### Micro-CT analysis

Ex vivo Micro-CT scanning of hind paw specimens was performed using a Bruker SkyScan 1176 (Bruker Co., Belgium). Scanning parameters were set to 55 kVp/180 µA, isotropic voxel size 9 μm, exposure time 300 ms, and a total rotation of 180°. Cone-beam filtered back-projection (FBP) reconstruction was carried out in NRecon. In CTAn, the region/volume of interest (ROI/VOI) was delineated from the midline of the ankle joint to include the entire metapodium of the paw. Binary segmentation was performed using either a fixed threshold or the Otsu method. Bone structural parameters, including BV/TV, Tb.Th, Tb.N, Tb.Sp, and Ct.Th, were quantified, and 3D surface renderings were exported for visualization. To ensure comparability, all samples were scanned with consistent orientation and parameter settings.

### H&E staining

Tissues were fixed in 4% paraformaldehyde at 4 °C for 24 h, dehydrated through an ethanol gradient, embedded in paraffin, and sectioned at 4 μm for skin or 8 μm for bone. Prior to embedding, bone specimens were decalcified in 10% EDTA (pH 7.4) at 4 °C for 21 days with the solution refreshed every other day. Histopathology was assessed on sections stained with hematoxylin and eosin (H&E).

### TRAP staining

Ankle joints were harvested, fixed in 4% paraformaldehyde at 4 °C for 24 h, and decalcified in 10% EDTA (pH 7.4) for 6–8 weeks with routine solution replacement. Specimens were then paraffin‑embedded, and 5 μm sagittal sections were prepared. For tartrate‑resistant acid phosphatase (TRAP) staining, sections were deparaffinized, rehydrated, and processed with TRAP working solution (P0332, Beyotime, China) containing naphthol AS‑BI phosphate and Fast Red Violet in the presence of 50 mM sodium tartrate, following the manufacturer’s protocol. TRAP‑positive multinucleated osteoclasts (≥ 3 nuclei) along the talar and tibial bone surfaces were counted and expressed as number per bone perimeter.

### Safranin O staining

Proteoglycan depletion and cartilage status in ankle joints were examined using Safranin O/Fast Green. Following deparaffinization and rehydration, 5 μm sagittal sections were stained in sequence with Weigert’s iron hematoxylin (5 min), 0.02% Fast Green (5 min), 1% acetic acid (10 s), and 0.1% Safranin O (5 min). Slides were then dehydrated, coverslipped, and imaged by light microscopy. Cartilage degeneration was graded semi‑quantitatively by two blinded evaluators using the Osteoarthritis Research Society International (OARSI) scoring criteria.

### Statistical analysis

Data are reported as mean ± standard deviation. Data were analyzed by one-way ANOVA and post-hoc multiple comparisons, *ns* indicates no statistically significant difference, *P < 0.05, **P < 0.01, ***P < 0.001.

## Supplementary Information

Supplementary Material 1

## Data Availability

The data that support the findings of this study are available from the corresponding author upon reasonable request.

## Declarations

### Declaration of AI

The author team independently developed this manuscript and employed ChatGPT exclusively for linguistic improvement after draft completion. Following this assistance, the authors reviewed and edited all content, accepting full responsibility for the final publication.
