# Supplementary material for: In-situ RhoA editing via heparinylated LNP-microsphere system for rheumatoid arthritis treatment
Source: J Nanobiotechnology. 2026 Jan 23;24:166. doi: 10.1186/s12951-026-04040-x (PMC12911233; doi:10.1186/s12951-026-04040-x)
Supplement: Supplementary file 1 — Supplementary Material 1 [file 12951_2026_4040_MOESM1_ESM.docx]

**Supplementary Information of**

**In-situ RhoA Editing *via*** **Heparinylated LNP-microsphere System for Rheumatoid Arthritis Treatment**

Yingchun Zhu ^a,1^, Lei Wang ^b,1^, Yingying Wei ^e,1^, Guanrong Li ^a^, Zheyuan Shi ^a^, Dianqing Wang ^a^, Qiang Wang ^b^, Liheng Wang ^c,d^*, Weibing Si ^c,d^*, Xing Yang ^c,d^*

a Department of Orthopaedic Surgery, The First Affiliated Hospital of Ningbo University, Ningbo 315010, P. R. China.

b Department of Orthopedics, The First Affiliated Hospital of Wannan Medical College, Yijishan Hospital of Wannan Medical College, Wuhu 241001, P. R. China.

c Department of Orthopedics, The Affiliated Suzhou Hospital of Nanjing Medical University, Suzhou Municipal Hospital, Suzhou 215000, P. R. China.

d Gusu School of Nanjing Medical University, Suzhou 215000, P. R. China.

e Department of Orthopedics, Shanghai Key Laboratory for Prevention and Treatment of Bone and Joint Diseases, Shanghai Institute of Traumatology and Orthopaedics, Ruijin Hospital, Shanghai Jiao Tong University School of Medicine, Shanghai 200025, P. R. China.

^1^ These authors contributed equally to this work

* Corresponding authors: 153027399@qq.com (Liheng Wang), swb20021121@163.com (Weibing Si), xingyangsz@njmu.edu.cn (Xing Yang)

**Supplementary Tables**

**Table S1.** sgRNA sequences

| Name | Sequences |
| --- | --- |
| RhoA-SgRNA-1 | ACCGTGGGCACATAGACCTC |
| RhoA-SgRNA-2 | GGAATGACGAGCACACGAGA |
| RhoA-SgRNA-3 | CTGGATAAGAGAGAGGCCGC |

**Table S2.** qPCR primer sequences

| Name | Sequences (5’ - 3’) |
| --- | --- |
| RhoA-F | CTTCAGCAAGGACCAGTTCCCA |
| RhoA-R | GGCGGTCATAATCTTCCTGTCC |
| IL-1β-F | TGGACCTTCCAGGATGAGGACA |
| IL-1β-R | GTTCATCTCGGAGCCTGTAGTG |
| TNF-α-F | GGTGCCTATGTCTCAGCCTCTT |
| TNF-α-R | GCCATAGAACTGATGAGAGGGAG |
| IL-6-F | TACCACTTCACAAGTCGGAGGC |
| IL-6-R | CTGCAAGTGCATCATCGTTGTTC |

**Supplementary Figures**


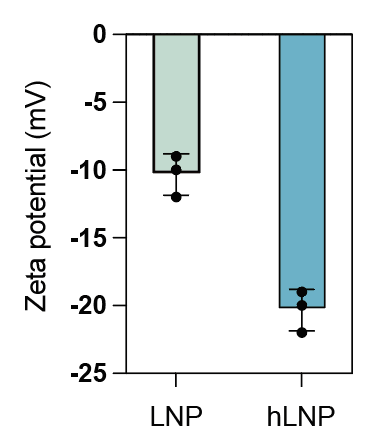


**Figure S1.** Zeta potential data for LNPs and hLNPs.


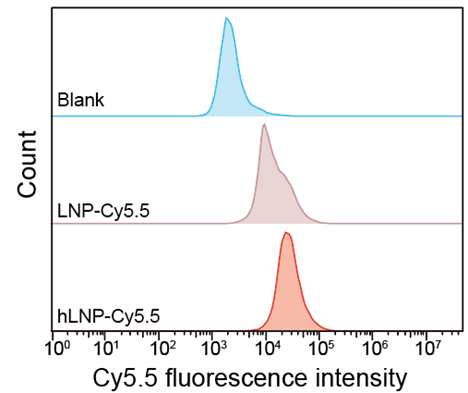


**Figure S2.** Cellular uptake in Raw264.7 cells after incubation with LNPs (LNP-Cy5.5) and hLNPs (hLNP-Cy5.5) formulated with Cy5.5-mRNA. LNPs or hLNPs were preincubated with TNF‑α (1,000 pg/mL) in complete medium for 2 h.


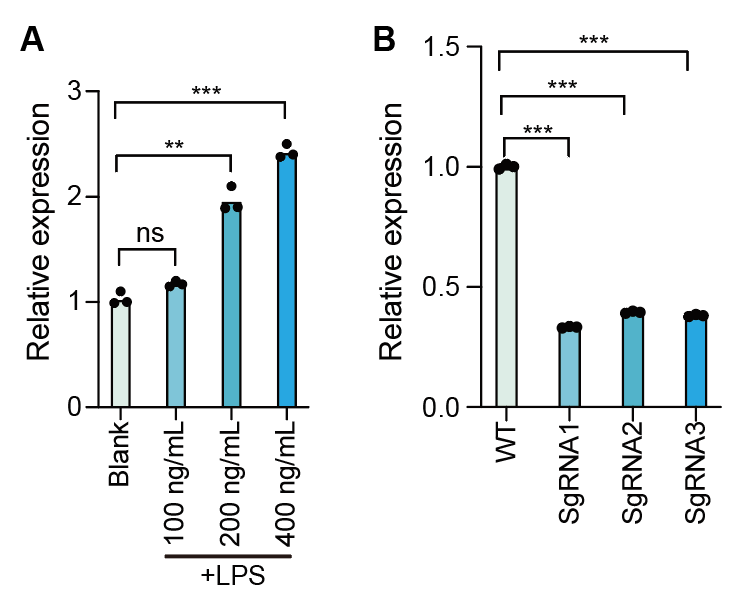


**Figure S3.** Semi-quantitative analysis of the Western blots in Figures 3A (A) and 3E (B).


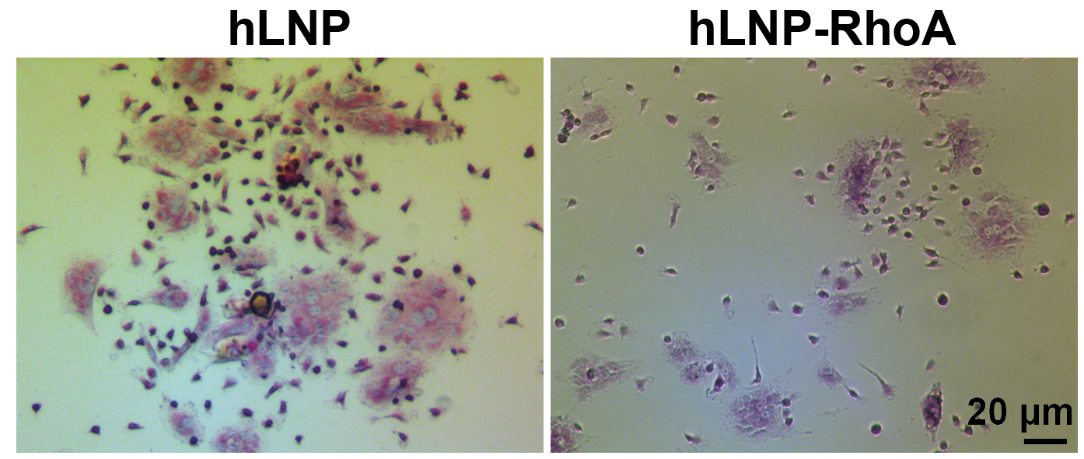


**Figure S4.** Following RhoA editing with hLNP‑RhoA, TRAP staining revealed a marked reduction in multinucleated osteoclast fusion in Raw264.7 cells after RANKL‑induced osteoclast differentiation


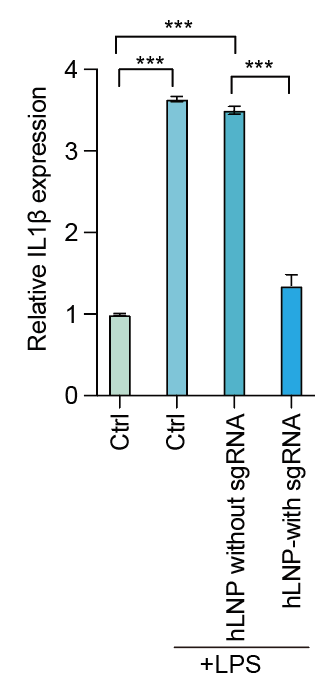


**Figure S5.** IL-1β mRNA expression in Raw264.7 cells measured by qPCR after treatment with RhoA−/− CRISPR hLNPs with or without sgRNA


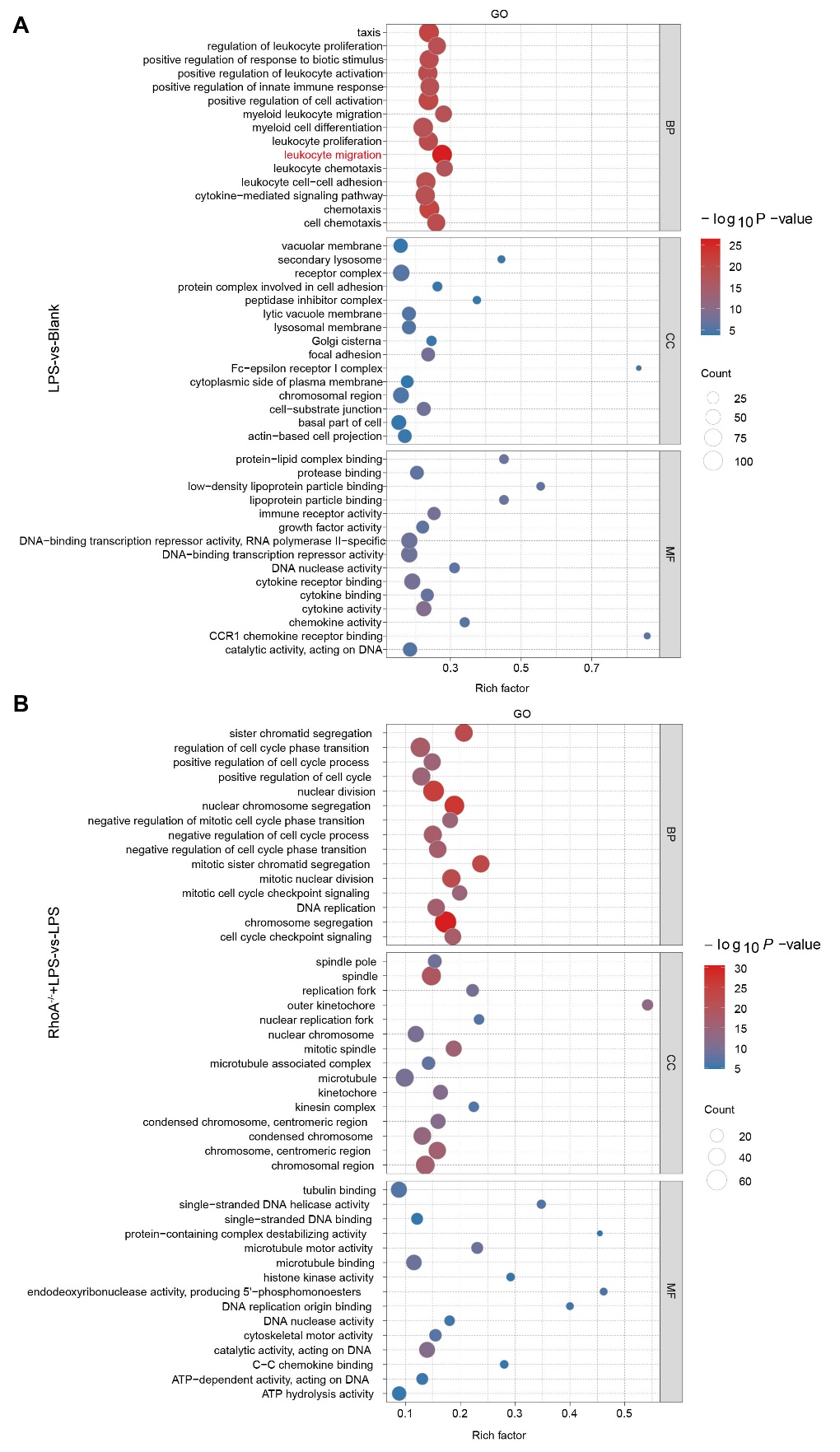


**Figure S6.** GO enrichment analysis reveals the regulatory role of RhoA deficiency in the LPS-induced response: (A) Differentially expressed genes in LPS vs. Blank are primarily enriched in immune cell activation, inflammatory cytokine production, chemotaxis, and cytokine receptor binding. (B) Differentially expressed genes in RhoA^⁻/⁻^ + LPS *vs.* LPS are mainly enriched in cell cycle, DNA replication, chromosome segregation, and microtubule-related functions.


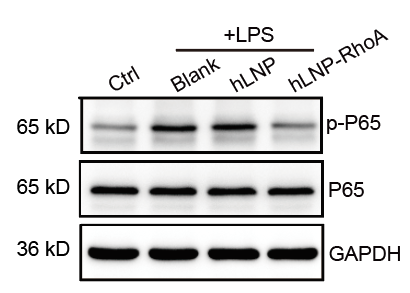


**Figure S7.** Phosphorylation levels of P65 (p-P65) in Raw264.7 cells after editing with hLNP‑RhoA (hLNP‑RhoA−/−), with unedited hLNPs serving as the control.


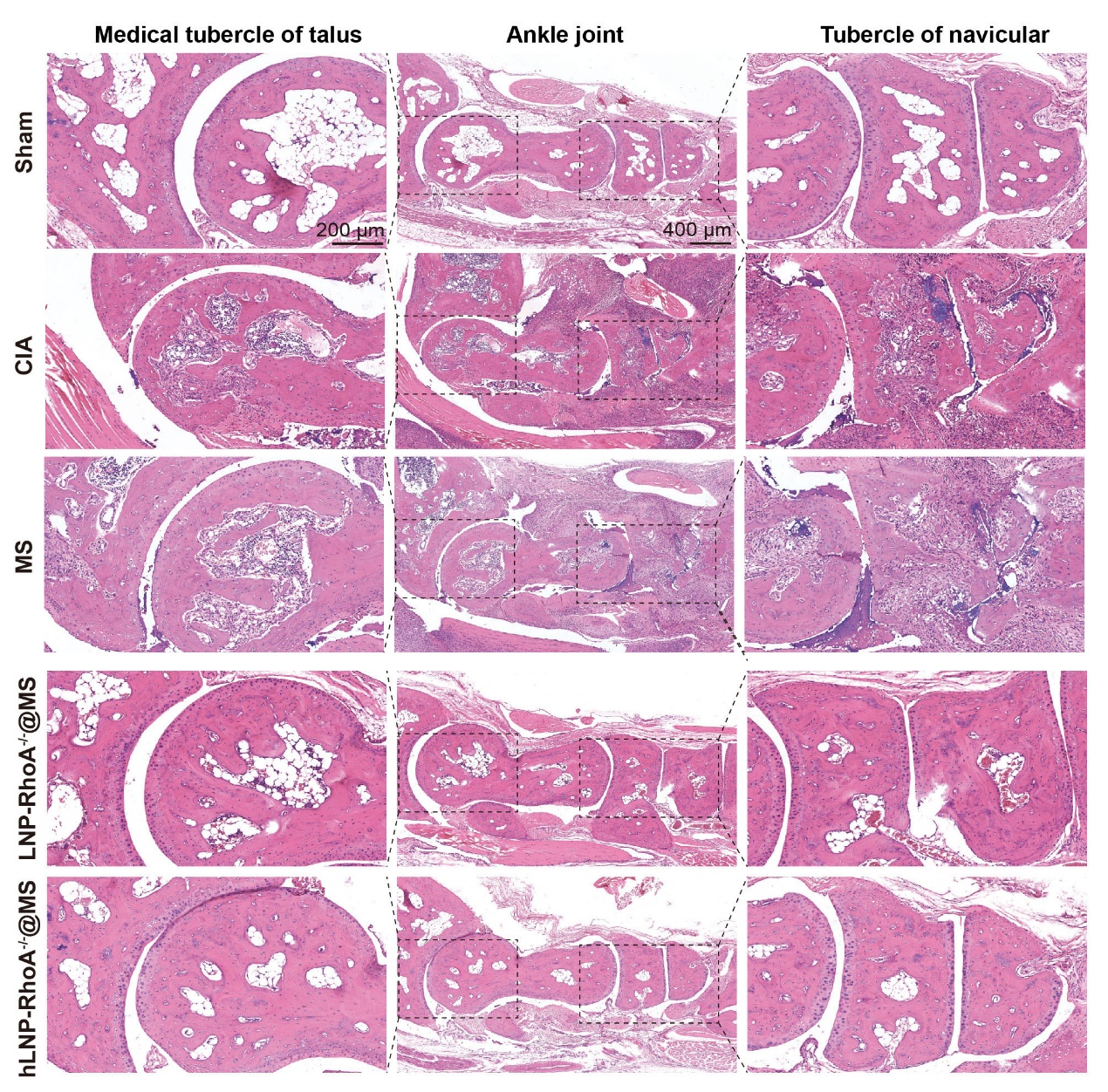


**Figure S8.** H&E staining of ankle joints.


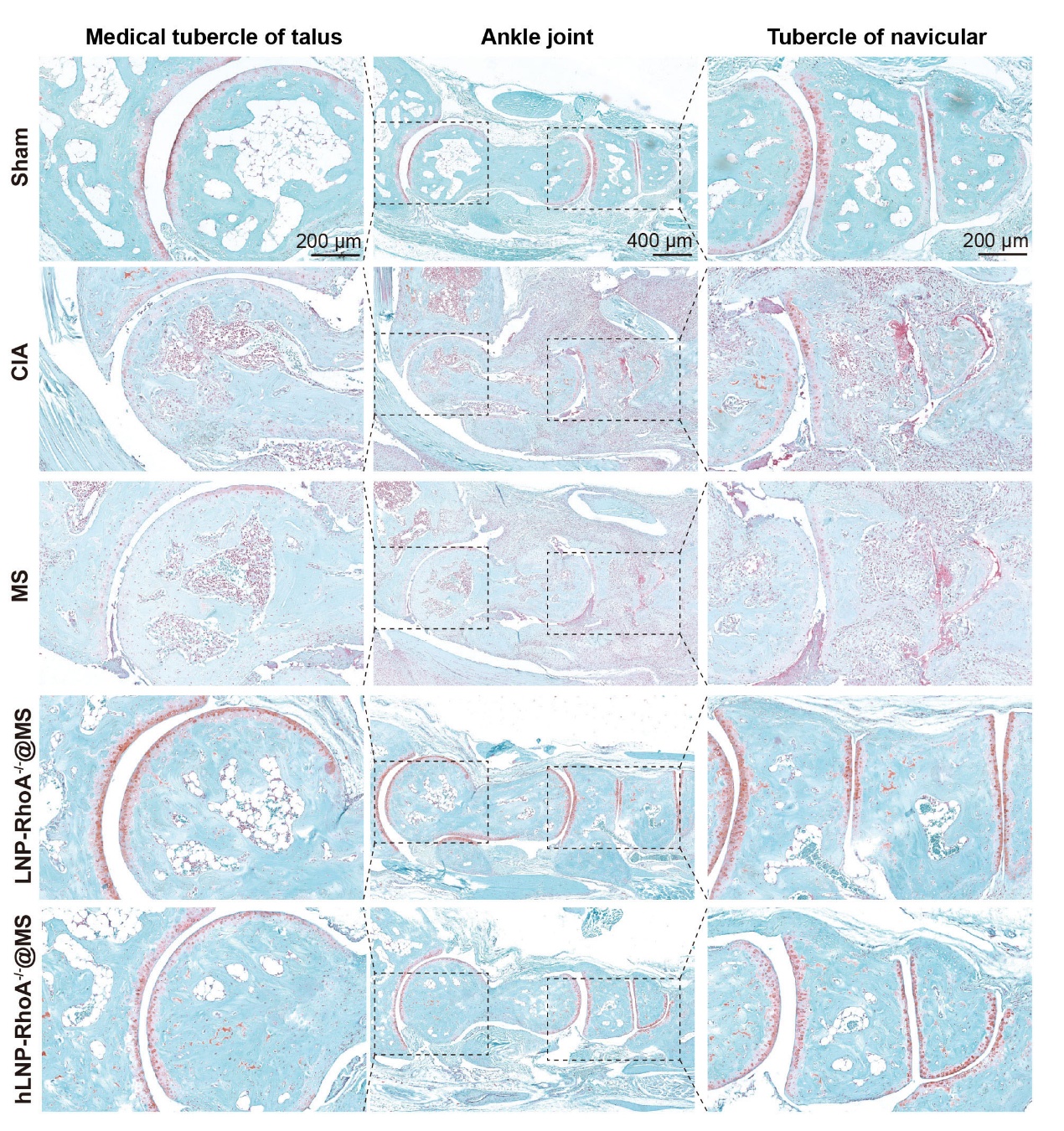


**Figure S9.** Safranin O staining of ankle joints.
